# The role of red cell distribution width in inflammatory bowel disease evaluation: a comprehensive systematic review and meta-analysis

**DOI:** 10.1186/s12876-026-05156-y

**Published:** 2026-07-27

**Authors:** Yasser Khalil, Mohamed A. Elhodhod, Mohammed Barghash, Mark Zaki, Abdelbaki Idriss Ibrahim, Mohamed Elserougi, Ahmed Ghanem, Khaled Ashraf Mohamed, Florian Gebauer, Ahmed Abdelsamad

**Affiliations:** 1 Faculty of Medicine, Helwan University, Cairo, Egypt; 2 Faculty of Medicine, Menoufia University, Menoufia, Egypt; 3 Department of General Surgery, North Manchester General Hospital, Manchester University Foundation Trust , Manchester, England; 4 Faculty of Medicine, Misr University for Science and Technology , 6th of October City, Egypt; 5 Cairo University Hospitals, Cairo University, Cairo, Egypt; 6 Faculty of Medicine, Cairo University, Giza, Egypt; 7 Gynecologist resident at Ras El Ten Hospital, Alexandria, Egypt; 8 General surgery resident at Cairo University Hospitals, Cairo University, Cairo, Egypt; 9 Department of Surgery II, University of Witten/Herdecke, Witten, Germany; 10 Deputy head of surgical oncology, section head robotic surgery, Knappschaft Vest- Hospital, Recklinghausen, 45657 Germany

**Keywords:** Inflammatory bowel disease, Crohn’s disease, Ulcerative colitis, RDW, Red cell distribution width, IBD, CD, UC

## Abstract

**Background:**

Inflammatory markers are routinely used in the evaluation of inflammatory bowel disease (IBD). Red blood cell distribution width (RDW) has previously been proposed as a potential biomarker in the evaluation of IBD. This meta-analysis and systematic review challenges the utility of RDW in IBD evaluation.

**Methods:**

We conducted a systematic review and meta-analysis following PRISMA guidelines. A search strategy was formulated based on Medical Subject Headings (MeSH) terms and other relevant medical terms. A comprehensive search of PubMed, Web of Science, and Scopus was performed up to 4th of September 2025. Data were extracted for identifying the role of RDW in IBD detection and its association with disease activity. Statistical analysis employed a random-effects model, with subgroup analysis performed according to different levels of disease activity.

**Results:**

RDW was significantly elevated in patients suffering from Crohn’s disease (CD) (MD = 2.2, *p* < 0.001) and ulcerative colitis (UC) (MD = 1.36, *p* < 0.001) compared to healthy controls. Both diseases showed significantly higher RDW in active disease versus remission (CD: MD = 1.29, *p* < 0.001; UC: MD = 1.11%, *p* < 0.001). For differentiating active CD from remission, an RDW cut-off > 14% showed a sensitivity of 0.86 and specificity of 0.78 (AUC = 0.83). RDW levels were also significantly higher in active CD compared to active UC (MD = 0.59, *p* < 0.005).

**Conclusion:**

RDW levels were markedly increased in patients with IBD compared to healthy controls. Furthermore, RDW showed a consistent association with both disease activity and severity. Given its wide availability, low cost, and reliable diagnostic performance, RDW could represent a practical and valuable biomarker for evaluating inflammatory bowel disease.

**Supplementary Information:**

The online version contains supplementary material available at 10.1186/s12876-026-05156-y.

## Introduction

Inflammatory bowel disease (IBD) comprises two main phenotypes—ulcerative colitis (UC) and Crohn’s disease (CD)—characterized by chronic, immune-mediated inflammation of the gastrointestinal tract with relapsing–remitting courses [1]. The population burden is substantial and rising: prevalence now exceeds ~ 0.3% in North America, Oceania, and much of Europe, translating to roughly 2.5–3 million affected individuals in Europe alone, with incidence expanding in newly industrialized regions [2, 3].

Diagnosis and treatment are complex and multidisciplinary, requiring integration of clinical assessment with endoscopic, histologic, laboratory, and imaging findings, followed by individualized, treat-to-target therapy [4, 5]. Earlier recognition of IBD is associated with timelier therapy, improved disease control, and easier downstream management—principles embedded in contemporary European (ECCO) guidance [5–7].

Multiple complementary tools are used to establish a diagnosis and monitor activity [8]. Colonoscopy with biopsy remains the reference standard; cross-sectional imaging (MR enterography, CT enterography, and intestinal ultrasound) delineates the extent and complications; and laboratory biomarkers such as C-reactive protein (CRP) and erythrocyte sedimentation rate (ESR) track systemic inflammation [4, 9]. Fecal calprotectin, a non-invasive neutrophil-derived marker, reliably discriminates IBD from functional disorders and reduces unnecessary endoscopy, especially in acute phases, with strong evidence for diagnostic accuracy and cost-effectiveness [10, 11].

Beyond these tests, the role of red cell distribution width (RDW)—an inexpensive, routinely reported index of anisocytosis influenced by inflammation and iron-restricted erythropoiesis has gained attention in IBD [12, 13]. Observational studies consistently report higher RDW in IBD versus healthy controls and in active versus remission states, with signals that RDW may help differentiate activity [13] and, in some series, distinguish CD from UC; however, cut-offs vary, study designs are heterogeneous, and results are not uniform across settings [11, 14, 15]. These uncertainties warrant formal evidence synthesis.

Accordingly, we conducted a comprehensive PRISMA-compliant, PROSPERO-registered systematic review and meta-analysis to evaluate RDW as a biomarker in diagnosing IBD. We compared RDW values (i) between IBD and healthy populations, and (ii) across activity states (active vs. remission) within CD and UC, and assessed diagnostic performance. By synthesizing disparate studies and exploring sources of heterogeneity, our objective was to clarify the utility and practical thresholds of RDW for disease assessment in UC and CD and to position RDW alongside existing laboratory and imaging tools in routine care, while RDW shows promise, it is essential to evaluate its performance as a supplementary marker rather than a replacement for established clinical and endoscopic modalities.

## Methods

### Search strategy

This systematic review and meta-analysis were conducted according to the Preferred Reporting Items for Systematic reviews and Meta-Analyses (PRISMA) checklist [16], and the principles of the Cochrane Handbook for systematic reviews of interventions version 6.2 [17]. The study protocol was registered in the PROSPERO international registry with a registration number of (CRD420251140416). The study was based on the following question: Can Red Cell Distribution Width (RDW) be used as a biomarker in patients suffering from Inflammatory Bowel Diseases (IBD), and can it be utilised to detect the disease severity in such patients?

**Fig. 1:**
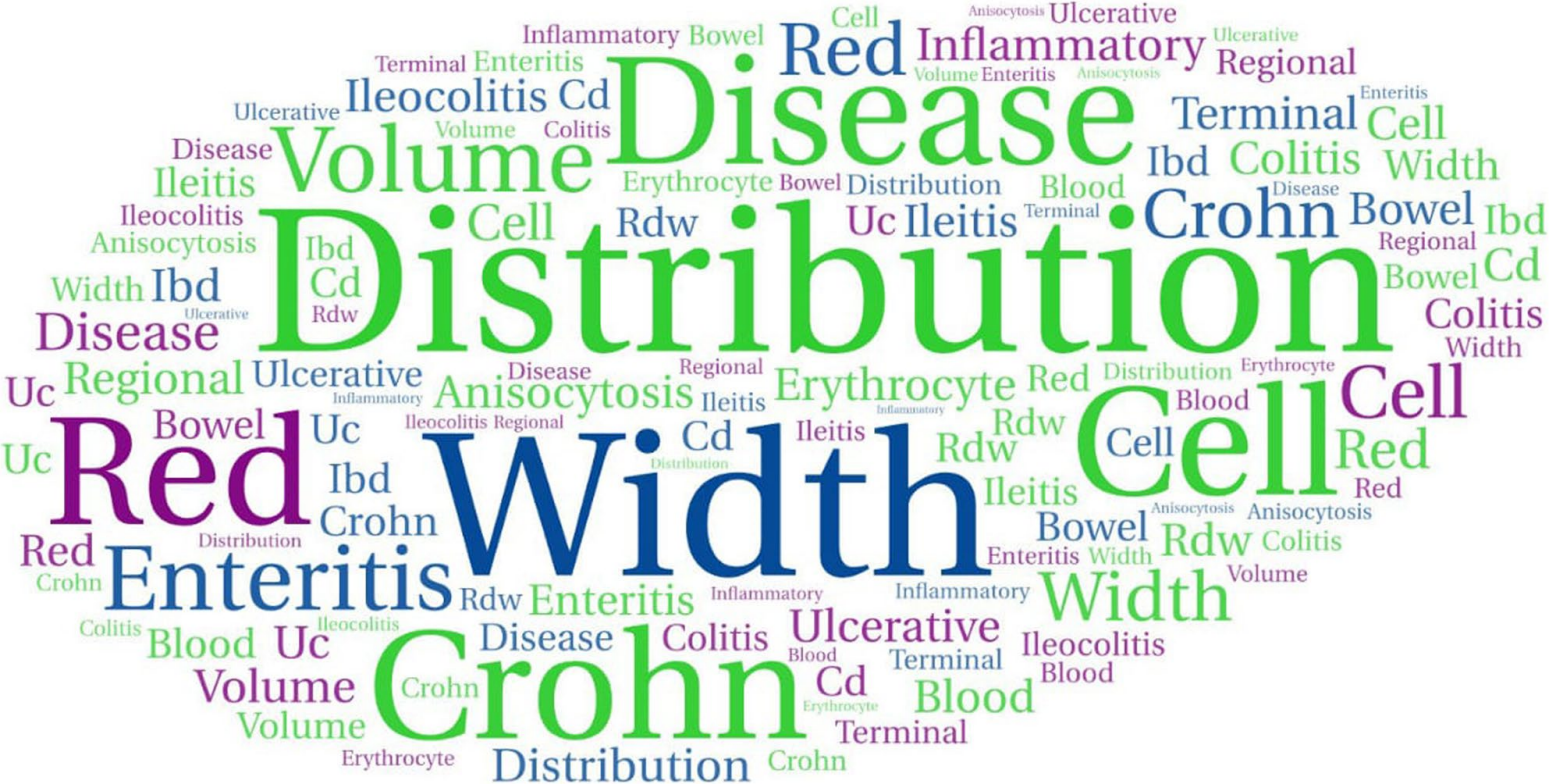
Word cloud

**Fig. 2:**
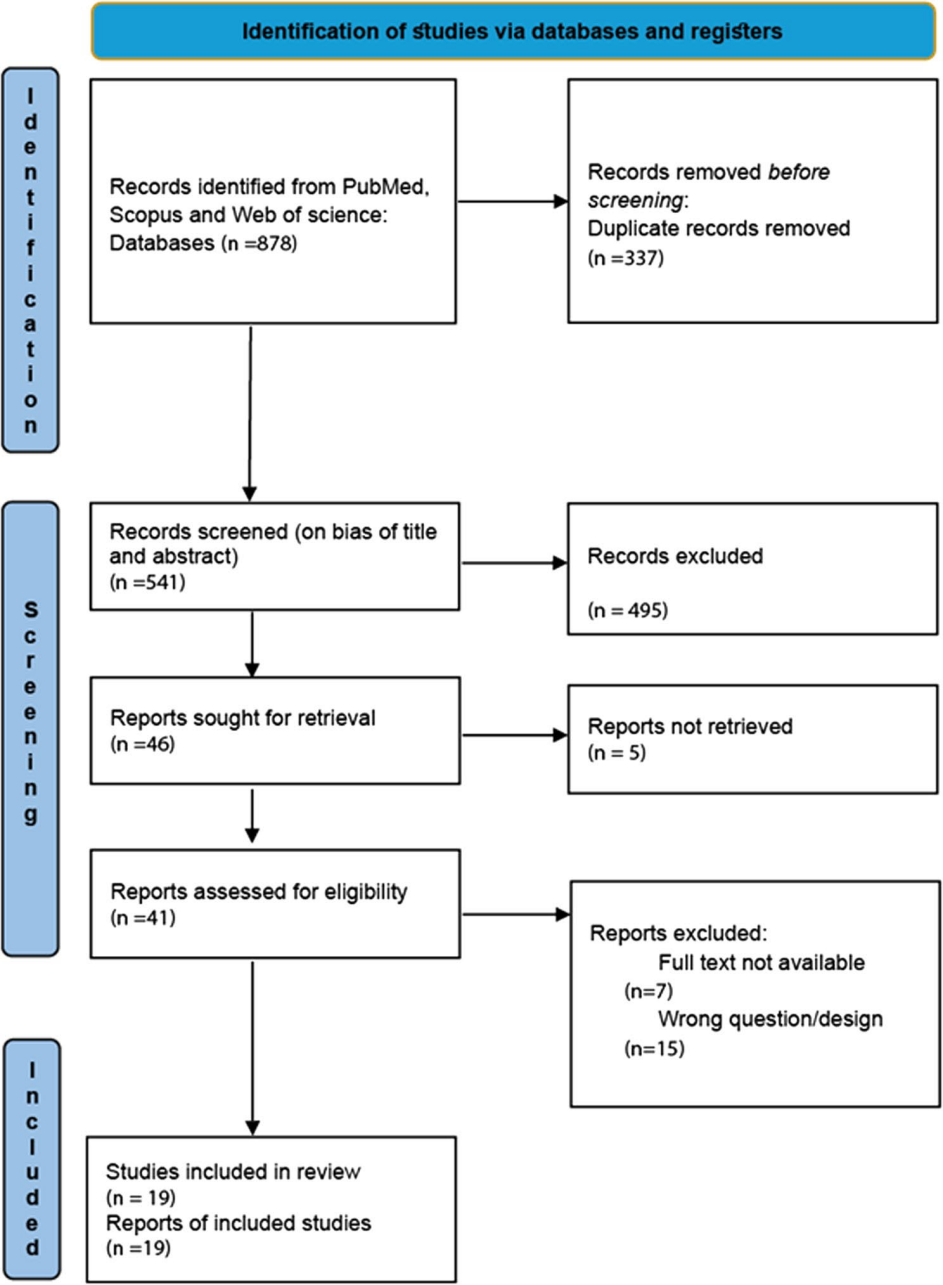
PRISMA flow chart

A search strategy was formulated based on Medical Subject Headings (MeSH) terms and other relevant medical terms from relevant studies, such as “inflammatory bowel disease”, “Crohn’s disease”, “Ulcerative colitis”, and “RDW”. Boolean operators such as AND as well as OR were used to combine the MESH terms to formulate the following search strategy: (Anisocytosis OR red cell distribution width OR RDW OR red blood cell distribution width OR erythrocyte volume distribution width OR red cell volume distribution width) AND (inflammatory bowel disease OR ulcerative colitis OR Crohn disease OR Crohn’s disease OR Crohn’s enteritis OR regional enteritis OR ileocolitis OR terminal ileitis OR IBD OR CD OR UC). Fig. 1 illustrates a word cloud that visually represents the search terms and their relevance, highlighting the main focus of the present systematic review. The literature search was conducted on the following major databases: PubMed, Web of Science, and Scopus up to 4th of September 2025. The process of article extraction is summarised in Fig. 2. The full search strategy and number of results retrieved from each database are presented in Supplementary Table 1.

### Eligibility criteria

The extracted articles were rigorously scrutinised to include the studies measuring RDW values in IBD patients, including active and/or remission states. To minimize publication bias, full-text articles published in peer-reviewed journals, as well as relevant conference abstracts, letters to the editor, or reports, were considered for inclusion in accordance with the Cochrane Handbook recommendations [17]. Article authors were contacted for any missing information. No restrictions or search filters were applied in terms of language, study design, or year of publication. On the other hand, we excluded the studies that didn’t include RDW values in relation to IBD, studies that didn’t include IBD, animal studies, duplicates, case reports, narrative reviews, and case series.

### Data extraction

Three authors (Y.K., M.A.E., and M.Z.) independently carried out the screening review, which began with a review of titles and abstracts, followed by a review of full texts and references. A third independent author resolved any disagreements between the three authors (K.A.). Studies were assessed based on the predefined inclusion and exclusion criteria. Two authors (M.E. and A.G.) collected patients’ characteristics, duration of IBD, and diagnostic tools.

### Quality assessment

The quality of the included studies was independently evaluated by two reviewers (M.Z. and A.I.). A third independent author resolved disagreements between the two authors (K.A.). For cross-sectional studies, we used the National Institutes of Health (NIH) quality assessment tool [18], which considered the clarity of study aims, adequacy of the study population, accuracy of exposure and outcome measurements, as well as adjustment for confounding factors. For observational cohort and case-control studies, we applied the Newcastle-Ottawa Scale (NOS) [19], which assessed three domains: study population selection, comparability of groups, and ascertainment of exposure or outcome. With a maximum score of 9, 7–9 indicated high quality, 4–6 demonstrated moderate quality, and 0–3 indicated low quality, as shown in Table 2. Overall study quality was categorised as good, fair, or poor. The methodological quality assessment of the present meta-analysis was comprehensively assessed by two reviewers using the AMSTAR 2 (A Measurement Tool to Assess Systematic Reviews 2) instrument [20], which examined 16 critical domains, including study selection, risk of bias assessment, adequacy of literature search, and appropriateness of statistical methods.

### Data analysis

Review Manager 5.4 [21] was used to calculate the mean difference and heterogeneity of RDW values between patients suffering from IBD vs. healthy subjects, patients with active IBD vs. healthy subjects, patients with active IBD vs. patients with IBD in remission, and patients suffering from Crohn’s disease (CD) vs. patients diagnosed with ulcerative colitis (UC). Forest plots were generated using a random effect model and 95% confidence intervals (CI). Between-study variance (τ²) was estimated using the DerSimonian-Laird method, which is the standard method used by Review Manager 5.4.

Subgroup analyses were conducted to the extracted data as follows: RDW levels in patients with CD vs. healthy subject, RDW levels in patients with active CD severity vs. healthy subject, RDW levels in patients with active CD vs. patients with CD in remission, and RDW levels in patients with active CD including severity stratification vs. patients with CD in remission, RDW levels in patients with UC vs. healthy subjects, RDW levels in patients with active UC including severity stratification vs. healthy subjects, RDW levels in patients with active UC vs. patients with UC in remission, RDW levels in patients with active UC including severity stratification vs. patients with UC in remission, and UC severity according to diagnostic tool used.

Meta-DiSc [22] was utilised to calculate sensitivity, specificity, positive likelihood ratio (PLR), negative likelihood ratio (NLR), diagnostic odds ratio (DOR), and the area under the Receiver Operating Characteristic (ROC) curve to reflect the accuracy of RDW in relation to IBD diagnosis and severity.

## Results

A total of 19 studies were included in the current meta-analysis with a pooled number of 3,780 patients. All outcomes were analysed to evaluate the role of RDW in assessing IBD diagnosis or activity. Results were presented in structured sections covering disease versus healthy controls, disease activity, sensitivity analyses, and diagnostic accuracy.

### Baseline characteristics of the included studies

Based on our analysis, the articles included were retrospective cohort studies (10 studies), cross-sectional observational studies (6 studies), prospective cohort studies (2 studies), and case case-control study (one study). Our calculations included a total of 3,780 patients, with 2,183 suffering from CD, 1,597 suffering from UC, and 1,147 healthy controls (reported in selected studies). The age of participants ranged from 27 to 60 years, with a male predominance, as shown in Table 1. The included studies utilized various diagnostic tools for inflammatory bowel diseases, including the Mayo score, Truelove-Witts, Harvey–Bradshaw Index (HBI), and endoscopic evaluations. However, the most utilised tools were the Crohn’s Disease Activity Index (CDAI) for CD and the Mayo score, as well as the Truelove-Witts for UC.

**Table 1:** Patients’ characteristics

Author	Year	Study design	Sample Size			Age			Gender(m/f)			IBD Type and Duration (Months)	RDW with IBD	Tools of Diagnosis
			UC (A/*R*)*	CD (A/*R*)*	Controls	UC	CD	Controls	UC	CD	Controls			
Arhan	2011	Retrospective study	105 (42/63)	60 (11/49)	43	39.7 ± 10.7	37.7 ± 13.2	35.3 ± 10.2	59/45	39/21	31//12	UC (76.7 ± 65.1), CD (86.3 ± 69.3)	Significantly elevated	Truelove-Witts scale (CDAI) and (EAI)
Cakal	2009	Retrospective study	74 (43/21)	22 (14/8)	20	39.8 ± 11.8	37.2 ± 10.6	37.6 ± 8.0	50/24	13//9	13//7	UC(60.8 ± 59.6), CD (39.8 ± 37.6)	Significantly elevated	Truelove-Witts scale, CDAI
J.Chen	2022	Retrospective study	183(118/65)	401 (202/199)		47	28		103/80	289/112		UC (15.79), CD (22.31)	Significantly elevated	Mayo score, CDAI
Yi-Han Chen	2020	Retrospective study	275 (177/98)	601 (302/299)		48	27		151/124	438/163		UC (15.83), CD (22.07)	Significantly elevated	Mayo score, CDAI
Hu	2015	Prospective study		100 (48/52)	102		33.2 ± 0.9	33.2 ± 0.8		67/33	67/35	CD	Significantly elevated	CDAI
Huang	2013	Retrospective study		68	22		35.5 ± 12.9	55.9 ± 15.7		43/25	12//10	CD (38.3 ± 45.7)	Significantly elevated	HBI
Ipek	2015	Retrospective study	310 (206/104)		104	45.6 ± 15.8		46.68 ± 13.9	132/74		59/45	UC	Significantly elevated	EAI, Rachmiletwiz activity index
Koc	2020	Retrospective study	98	34		40.8± 14.6	37.7 ± 12.5		57/41		20/14	UC (29.7 ± 45.9), CD (28.3 ± 39.9)	Significantly elevated	Mayo score, CDAI
Manesh	2012	Cross sectional observational cohort	96 (47/49)		51	35.26± 12.41		36.16± 11.52	54/42		27/24	UC (36)	Significantly elevated	DAI
Oliveira	2015	Cross sectional observational cohort		119 (20/99)			47 + 15.2			52/67		CD	Significantly elevated	CDAI
Oustamanolakis	2010	Cross sectional observational cohort	49 (11/38)	51 (9/42)	102	50.1	48.2	45.5	32/17	26/25	20/82	UC (105.6), CD (87.6)	Significantly elevated	European Crohn’s and Colitis Organization (ECCO) Evidence, Montreal Classification for disease phenotype, and CDAI
Rosseto-Welter	2022	Prospective study		16	28		41	39		5/11	9/19	CD	Significantly elevated	Histological, endoscopic and imaging criteria, CDAI
Song	2011	Retrospective Cohort	120(60/60)	101 (55/46)		45.3	37.3		83/18	83/37		UC, CD	Significantly elevated	Mayo score, CDAI
Tang	2015	Retrospective Cohort		130	130		33.4 ± 3.2	31.6 ± 1.2		62/68	67/63	CD	Significantly elevated	Double-balloon endoscopy and/or capsule endoscopy, CDAI
Vaghari-Tabari	2020	Retrospective Cohort	36	14	50	34.00 ± 10.20		36.00 ± 10.34				UC, CD	Significantly elevated	Colonoscopy and Histopathology
Xue	2023	Cross sectional observational cohort		303 (194/109)	293	28.6 ± 11.7		30.1 ± 12.3		216/87	190/103	CD	Significantly elevated	Consensus Opinion on the Diagnosis and Treatment of Inflammatory Bowel Disease (2018, Beijing), CDAI
Yesil	2011	Cross sectional observational cohort	61 (35/26)	56 (29/27)	44	40.3 ± 12	38 ± 10	38.9 ± 11	26/35	30/26	21/23	UC (45.6 ± 43.8), CD (36.6 ± 30.5)	Significantly elevated	Truelove-Witts or CDAI
Voudoukisa	2013	Cross sectional observational cohort	91	107	102	48	40.7		33/58	50/57		UC (84) ,CD (96)	Significantly elevated	CDAI and Simple Clinical Colitis Activity Index
Yalaki	2020	Case-control study	99 (63/36)		56	42.52		46.25	60/39		32 /24	UC	Significantly higher	Colonoscopy using EVIS LUCERA, ELITE CLV-290SL, Criteria of Truelove and Witts

### Risk of bias assessment

Quality assessment was performed using the Newcastle-Ottawa Scale (NOS) and NIH tool. Among cohort studies, NOS scores ranged from 5 to 8, indicating moderate to high quality as demonstrated in Table 2. One case-control study (Yalaki 2020) demonstrated a score of 5 in Table 3. Cross-sectional studies evaluated by the NIH tool generally demonstrated “fair” to “good” quality as shown in Table 4.

**Table 2:** Newcastle-Ottawa Scale cohort studies

	Selection				comparability	outcome			Total
	Representativeness of exposed cohort	Selection of non-exposed cohort	Ascertainment of exposure	Outcome not present at the start of the study		Assessment of outcomes	Length of follow-up	Adequacy of follow-up	
Arhan 2011	★	★	★	-	★	★	-	★	6
Cakal 2009	★	★	★	-	★	★	-	★	6
J.Chen 2022	★	★	★	★	★★	★	-	★	8
Yi-Han Chen 2020	★	★	★	-	★★	★	-	★	7
Hu 2015	★	★	★	★	★	★	-	-	6
Huang 2013	★	★	★	★	-	★	-	-	5
Ipek 2015	★	★	★	★	★	★	-	-	6
Koc 2020	★	★	★	★	★	★	-	-	6
Rosseto-Welter 2022	★	★	★	-	★★	★	-	-	6
Song 2011	★	★	★	-	★★	★	-	-	6
Tang 2015	★	★	★	-	★	★	-	-	5
Vaghari-Tabari 2020	★	★	★	-	★	★	-	★	6

**Table 3:** Newcastle-Ottawa Scale case-control studies

	Selection				Comparability	outcome			Total
	Case definition adequate	Representativeness of cases	Selection of control	Definition of controls		Assessment of exposure	Same method of ascertainment for cases and controls	non-response rate	
Yalaki 2020	★	-	-	★	★	★	★	-	5

**Table 4:** NIH quality assessment of included cross-sectional studies

	CK1	CK2	CK3	CK4	CK5	CK6	CK7	CK8	CK9	CK10	CK11	CK12	CK13	CK14	Quality
Yeşil 2011	Y	Y	NR	Y	N	N	N	Y	Y	N	Y	NR	N	Y	GOOD
Xue 2023	Y	Y	NR	Y	N	N	N	Y	Y	N	Y	NR	N	CD	FAIR
Oustamanolakis 2010	Y	Y	NR	Y	N	N	N	Y	Y	N	Y	NR	N	N	FAIR
Oliveira 2015	Y	Y	NR	Y	N	N	N	Y	Y	N	Y	NR	N	N	FAIR
Manesh 2012	Y	Y	NR	Y	N	N	N	Y	Y	N	Y	NR	N	Y	GOOD
Voudoukis 2013	Y	Y	NR	NR	N	N	N	Y	Y	N	Y	NR	N	Y	FAIR

### RDW levels and Crohn’s disease

#### Patients diagnosed with Crohn’s disease vs. healthy individuals

**Fig. 3:**
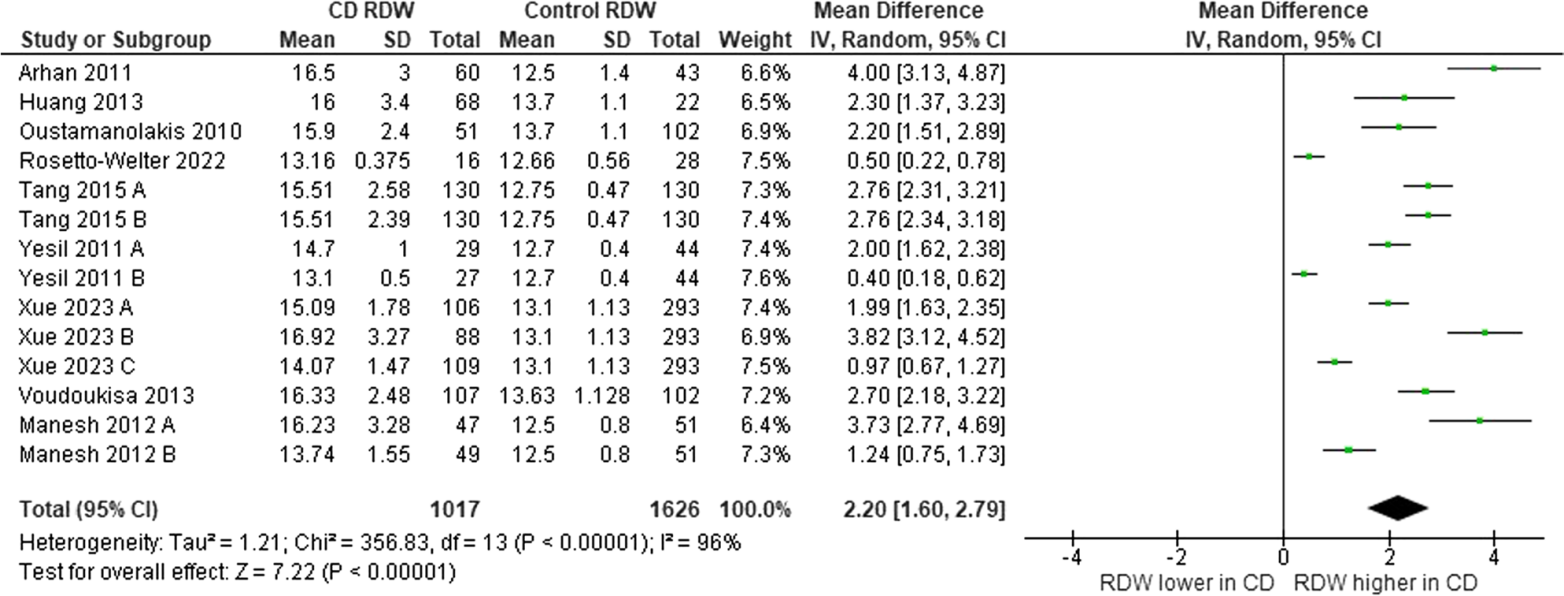
Patients diagnosed with CD vs healthy individuals

We included nine studies [13, 15, 23–29] comparing the RDW values in patients diagnosed with CD and healthy controls. Our calculations demonstrated significantly higher RDW values in patients suffering from CD (MD = 2.2; 1.6–2.79; *P* < 0.001). Due to significant heterogeneity observed among the included studies (I² = 96%, *P* < 0.001; Fig. 3), a leave-one-out sensitivity analysis was performed. This analysis did not meaningfully reduce heterogeneity, indicating that no single study disproportionately influenced the results.

#### Subgroup analysis of RDW levels in patients diagnosed with Crohn’s disease

##### Patient stratification according to disease activity vs. healthy individuals

**Fig. 4:**
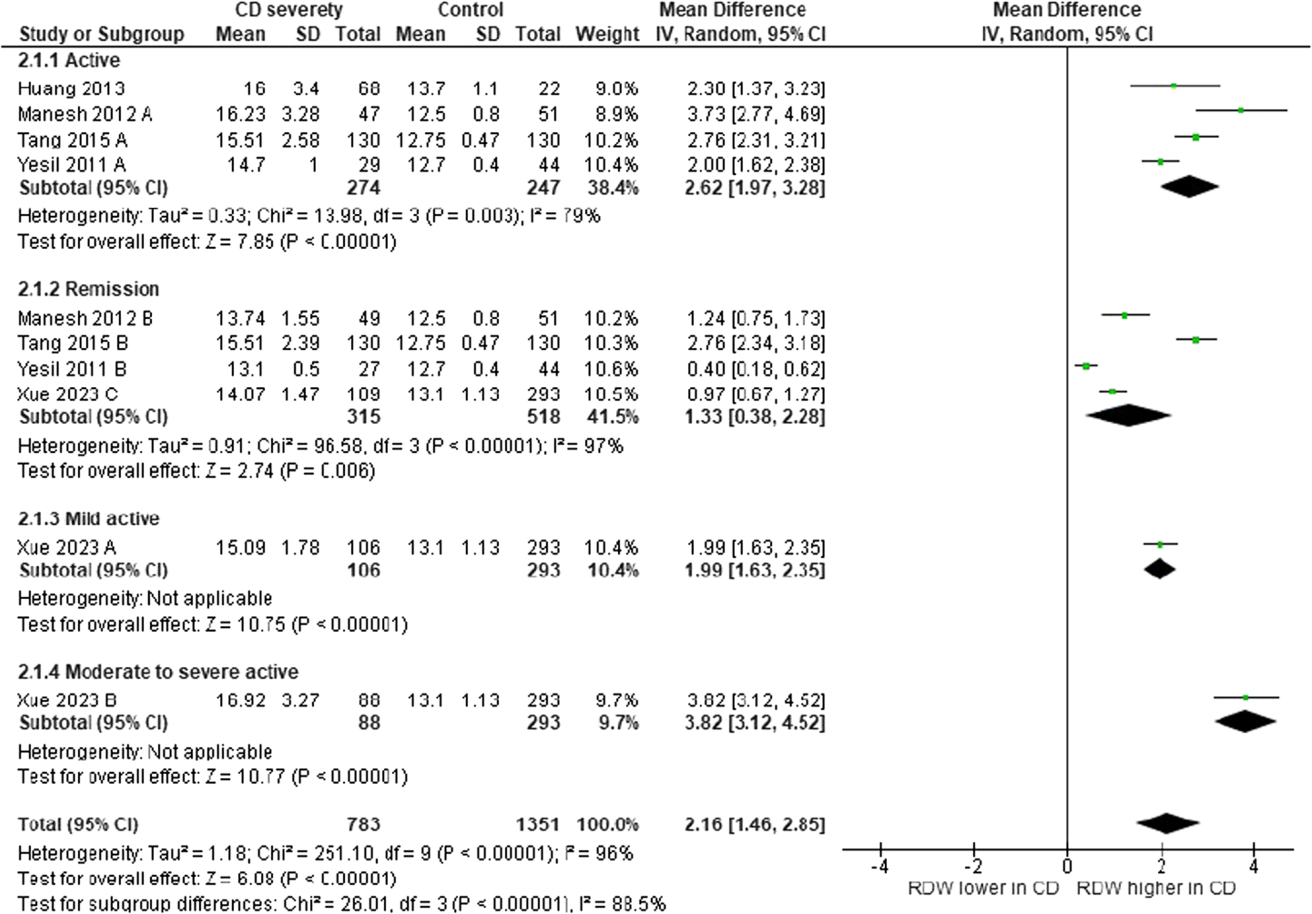
Patient stratification according to CD disease activity vs healthy individuals

Four studies [13, 23, 26, 29] documented RDW values in active CD patients versus healthy individuals. There were statistically significantly higher RDW values in active CD patients in comparison to healthy controls (MD = 2.62; 1.97–3.28; *P* < 0.001) with a statistically significant heterogeneity (I^2 = 79%, *P* = 0.003). On the other hand, four studies [13, 26, 27, 29] reported CD in remission state. Significantly higher RDW values were demonstrated in patients with CD remission state in comparison to healthy individuals (MD = 1.33; 0.38–2.28; *P* = 0.006) with a statistically significant heterogeneity (I^2 = 97%, *P* < 0.00001). One study [27] reported significantly high RDW levels in patients with mild CD activity (MD = 1.99; 1.63–2.35; *P* < 0.001). The same study indicated significantly higher RDW levels in patients suffering from CD with moderate to severe activity (MD = 3.82; 3.12–4.52; *P* < 0.001). That is highlighted in Fig. 4.

##### Patients suffering from active Crohn’s disease vs. individuals with Crohn’s disease in remission

**Fig. 5:**
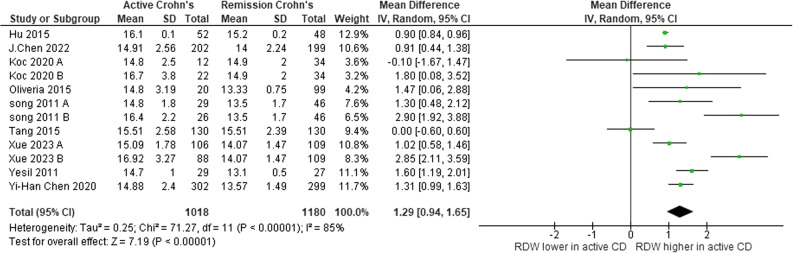
Patients with active CD vs patients in remission

Nine studies [13, 14, 26, 27, 30–34] compared the RDW values in patients suffering from active CD and patients in remission. According to our analysis, the RDW values were significantly higher in patients with active CD (MD = 1.29; 0.94–1.65; *P* < 0.001). However, as shown in Fig. 5, significant heterogeneity was observed (I^2 = 85%, *P* < 0.001).

**Fig. 6:**
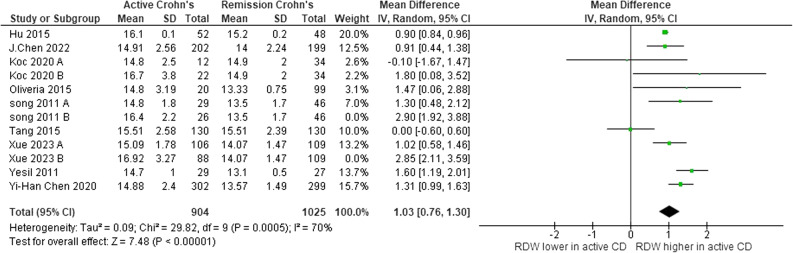
Leave one out sensitivity analysis of patients suffering from active Crohn’s disease vs individuals with Crohn’s disease in remission (Xue 2023 (B) and Song 2011 (B) excluded)

Three out of the nine studies [27, 32, 33] reported the severity of the disease activity according to CDAI (150 ≤ mild activity < 220; ≥220, moderate-to-severe activity) versus remission CD (remission < 150). Leave one out sensitivity analysis demonstrated that by excluding Xue 2023 (B), the heterogeneity decreased from 85% to 78%. The combined exclusion of Song 2011 (B) and Xue 2023 (B) further reduced the heterogeneity to 70% as shown in Fig. 6. The residual heterogeneity may be explained by the wide range of disease duration among patients, as well as the variability of the quality of the included studies, as outlined in our quality assessment table.

##### Patient stratification according to Crohn’s disease activity vs. patients in remission

**Fig. 7:**
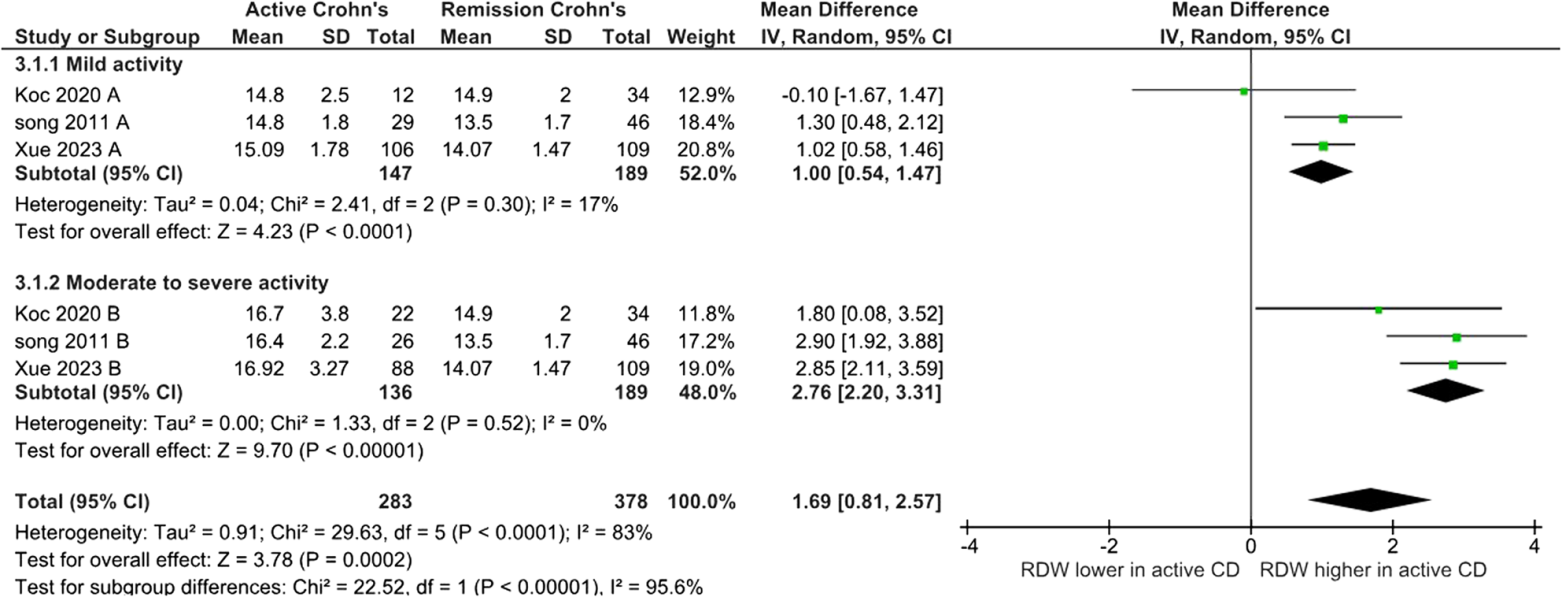
Patient stratification according to Crohn’s disease activity vs patients in remission

Three studies [27, 32, 33] examined RDW levels in patients suffering mild CD activity versus patients in remission. Significantly higher RDW levels were observed in mild CD activity (MD = 1.0; 0.54–1.47; *P* < 0.001). Those studies also compared RDW values in patients suffering from moderate to severe CD activity versus patients in remission. Again, significantly higher RDW levels were observed in patients with moderate to severe CD activity (MD = 2.76; 2.2–3.31; *P* < 0.001). That is highlighted in Fig. 7.

#### Diagnostic accuracy of RDW levels in Crohn’s disease

**Fig. 8:**
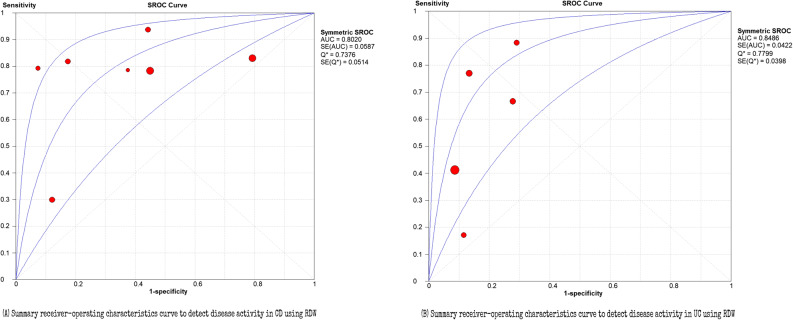
Summary receiver-operating characteristic curve to detect disease activity in IBD patients using RDW (Left ROC curve for CD patients. Right ROC curve for UC patients)

We pooled seven studies [13, 14, 26, 27, 30, 33, 35] reporting RDW diagnostic accuracy in differentiating between active and CD in remission. The disease activity was defined according to the CDAI. Our calculations demonstrated the sensitivity and the specificity of RDW in detecting CD severity as 0.8 (0.76–0.83) and 0.58 (0.53–0.62), respectively. The pooled DOR was 7.14 (2.85–17.94), while the PLR and NLR were 2.33 (1.41–3.87) and 0.37 (0.22–0.62), respectively as provided in Supplementary Figs. 7–11. The area under the curve (AUC) was calculated as 0.8 as shown in Fig. 8.

**Fig. 9:**
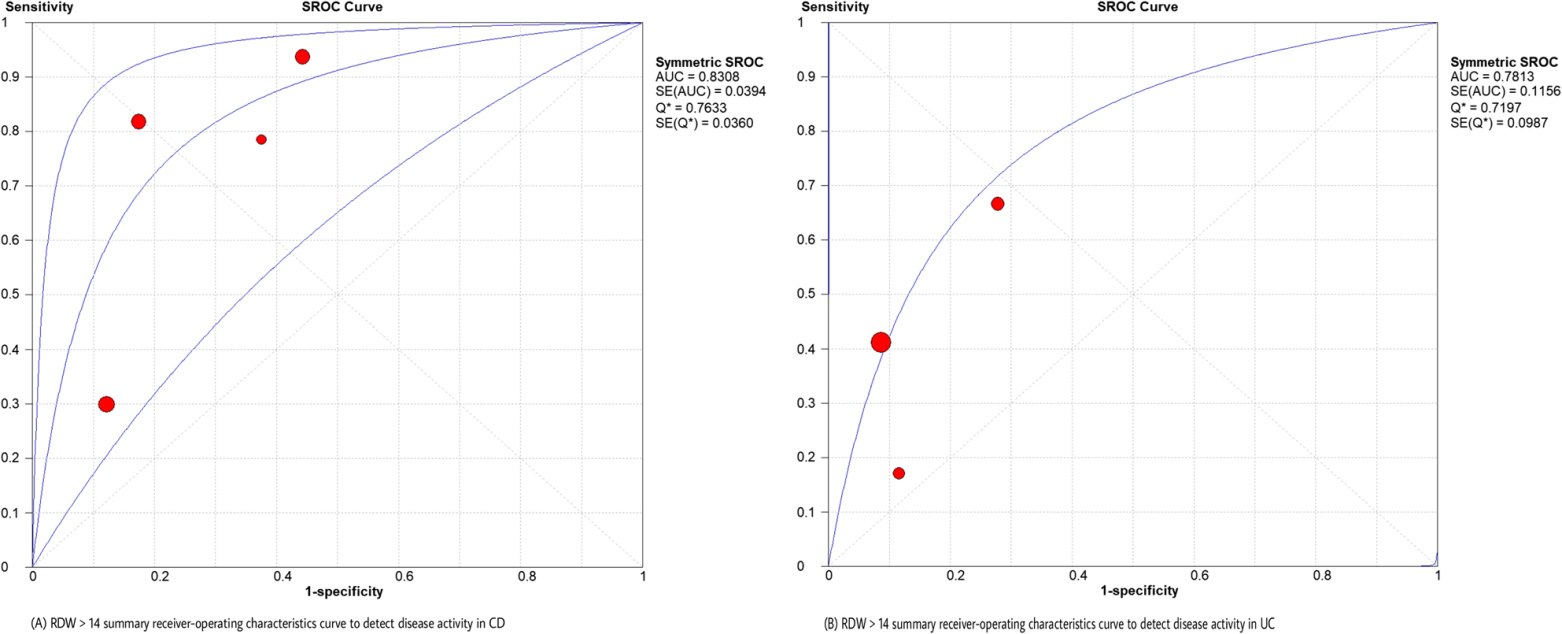
Summary receiver-operating characteristic curve of RDW > 14 to detect disease activity in IBD patients (Left ROC curve for CD patients. Right ROC curve for UC patients)

**Fig. 10:**
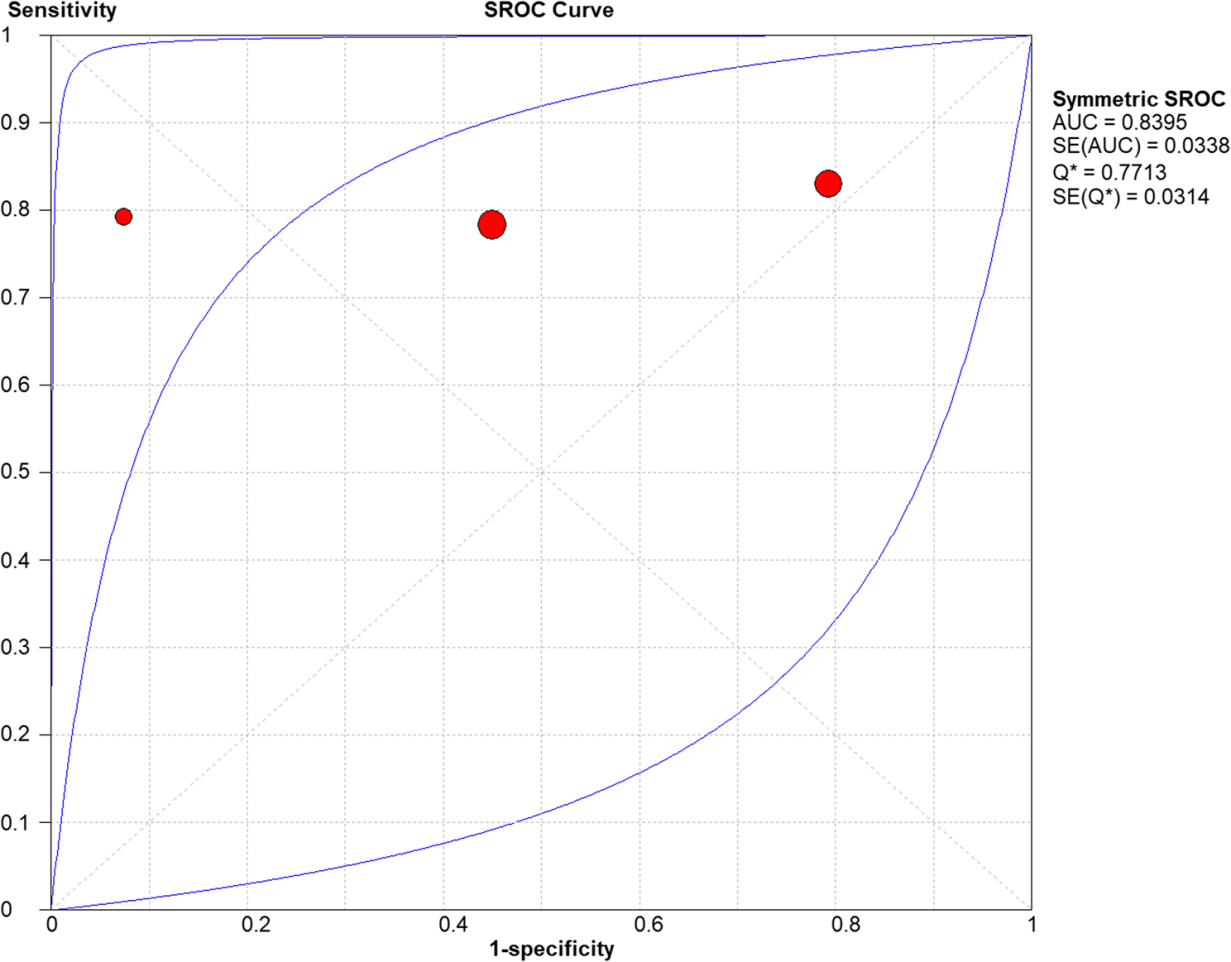
Summary receiver-operating characteristic curve of RDW < = 14 to detect disease activity in CD patients

Different studies used different RDW cut-off levels to identify active CD from remission CD, with cut-off values ranging from 13.25% to 16%. We proposed the RDW value > 14% to be used as a cut-off value to differentiate active CD from remission. utilised > s [14, 30, 33, 35] utilised > 14% as the RDW value cut-off. As such, sensitivity, specificity, and AUC were calculated as 0.78 (0.7–0.85), 0.78 (0.71–0.83), and 0.83, respectively (Fig. 9). By excluding Olivera 2015, the sensitivity increased to 0.86 (0.79–0.92) as shown in Supplementary Fig. 15.Three studies [13, 26, 27] utilised ≤ 14% as the RDW value cut-off. The summary receiver-operating characteristic (ROC) curve of RDW < = 14 to detect disease activity in CD patients is highlighted in Fig. 10.

### RDW levels and Ulcerative colitis

#### Patients diagnosed with ulcerative colitis vs. healthy individuals

**Fig. 11:**
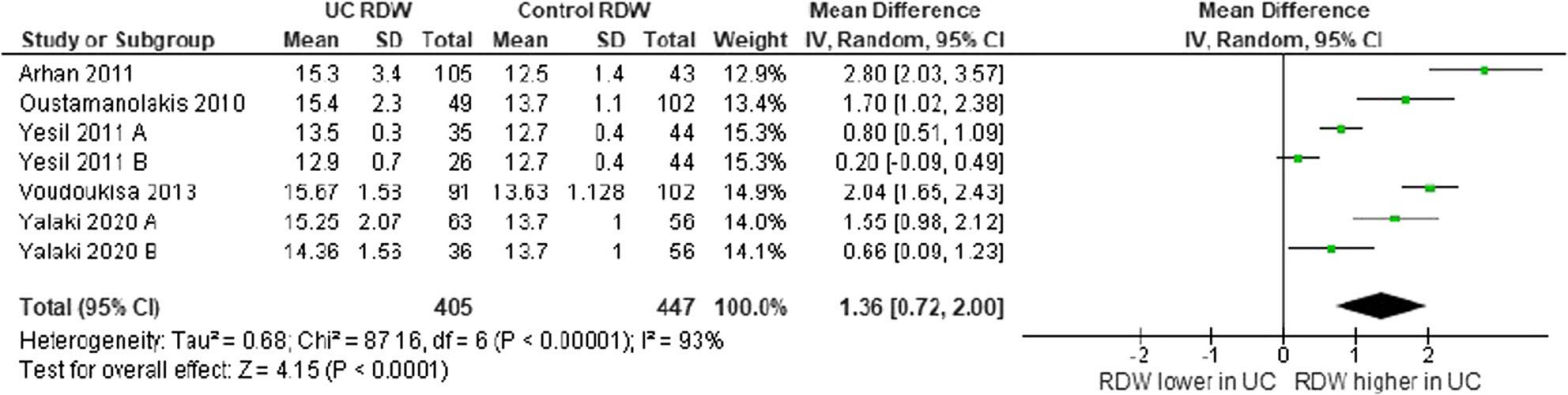
Patients diagnosed with UC vs. healthy individuals

We included five studies [13, 15, 24, 28, 36] to compare RDW values in patients suffering from UC and healthy controls. The RDW levels were significantly higher in UC patients (MD = 1.36; 0.72-2.0; *P* < 0.001) compared to healthy individuals. However, the heterogeneity was found to be statistically significant (I^2 = 93%, *P* < 0.001). That is highlighted in Fig. 11. Sensitivity analysis by exclusion of Yesil 2011(B) decreased the heterogeneity from 93% to 89% as shown in Supplementary Fig. 2. The remaining heterogeneity may be attributed to the variability of the included studies in terms of quality, as indicated in our quality assessment table.

Two studies [13, 36] reported the disease activity according to Truelove and Witts criteria. Patients were classified as mild, moderate, or severe depending on their daily bloody stool count, heart rate, hemoglobin, ESR, and body temperature. Moderate and severe disease classes were evaluated as active disease in comparison to healthy subjects.

#### Subgroup analysis of RDW levels in patients diagnosed with ulcerative colitis

##### Patient stratification according to disease activity vs. healthy individuals

**Fig. 12:**
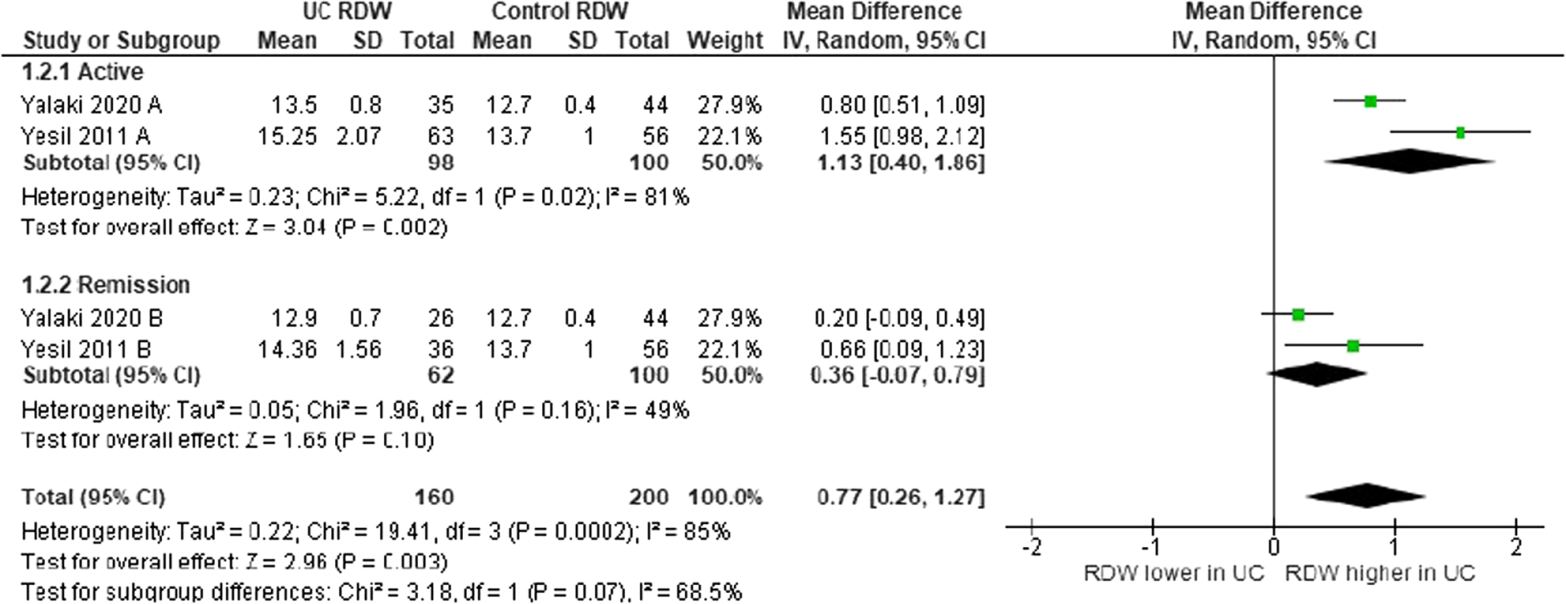
Patient stratification according to UC disease activity Vs healthy individuals

Two studies [13, 36] reported RDW levels in patients suffering from active UC vs. healthy individuals. Our calculations highlighted significantly higher RDW levels in patients diagnosed with active UC (MD = 1.13; 0.4–1.86; *P* = 0.002) with significant heterogeneity (I^2 = 81%, *P* = 0.02). Interestingly, in the same studies, insignificant RDW values were noted while patients were in a remission state compared to healthy individuals (*P* = 0.1). That is demonstrated in Fig. 12.

##### Patients suffering from active UC vs. individuals with UC in remission

**Fig. 13:**
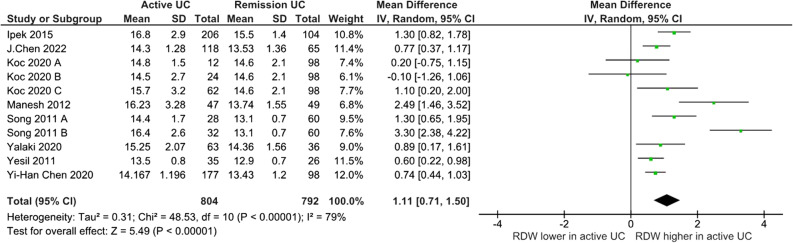
Patients with active Ulcerative colitis vs. patients in remission

We included eight studies [13, 29–32, 34, 36, 37] that compared the RDW values in patients with active UC with those in patients in remission. According to our analysis, the RDW values were significantly higher in patients with active UC (MD = 1.11; 0.71–1.5; *P* < 0.001). However, as demonstrated in Fig. 13, significant heterogeneity was observed (I^2 = 79%, *P* < 0.001). Five studies [29, 31–34] reported the disease activity using the Mayo score (remission if ≤ 3, mild activity if up to 6; ≥6 moderate-severe activity).

**Fig. 14:**
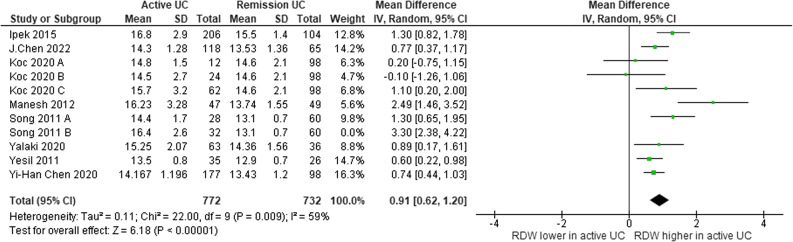
Leave one out sensitivity analysis of patients suffering from active UC vs. individuals with UC in remission (Song 2011 (B) excluded)

Sensitivity analysis by exclusion of Song 2011 (B) decreased the heterogeneity from 79% to 59% as shown in Fig. 14. The residual heterogeneity may be explained by the variability of tools utilised to diagnose UC, as well as the variability of the quality of the included studies, as outlined in our quality assessment table.

##### Patient stratification according to the utilised diagnostic tool for UC activity Vs patients in remission

**Fig. 15:**
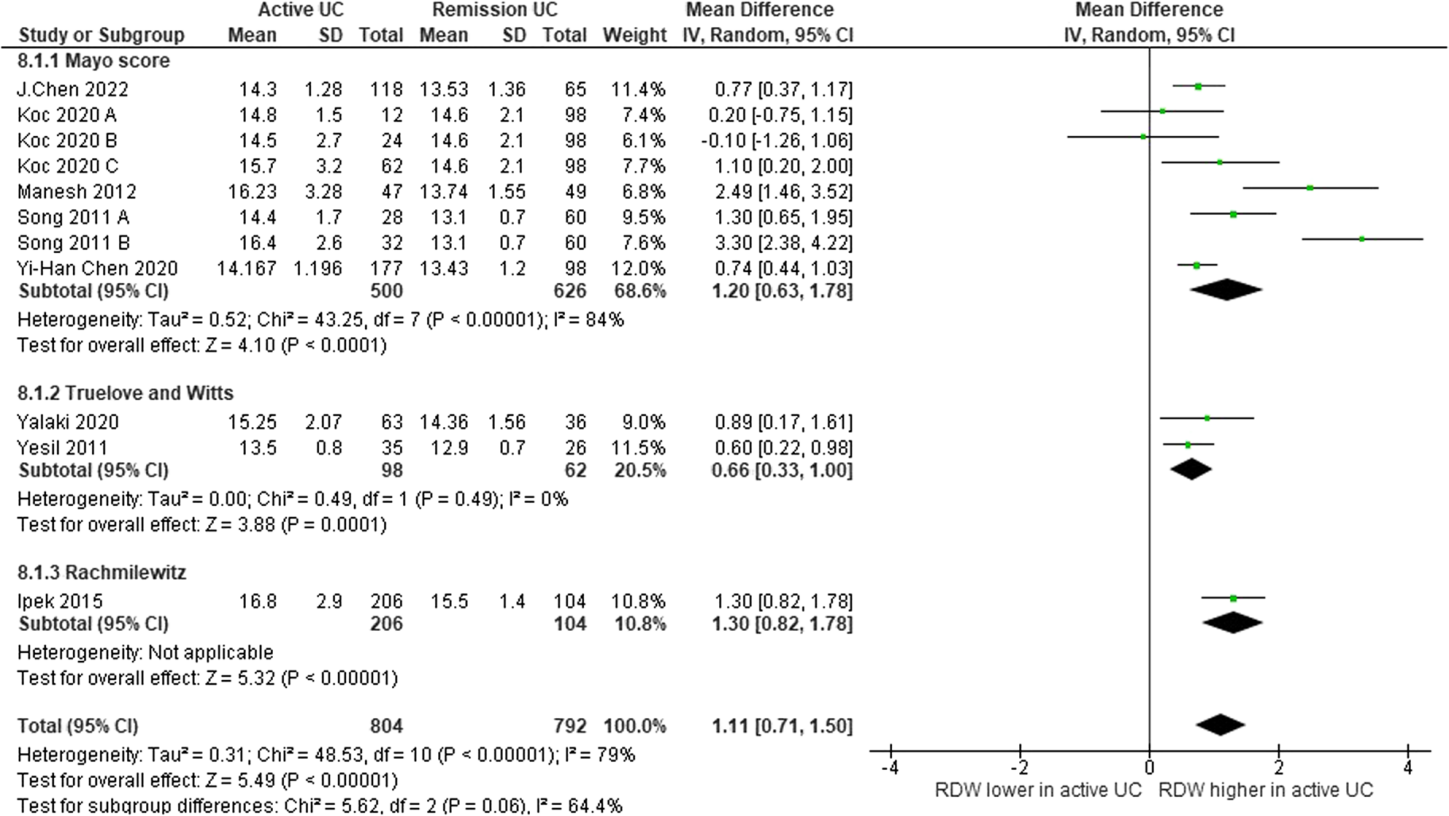
Patient stratification according to the utilised diagnostic tool for UC activity vs. patients in remission

Eight studies [13, 29–32, 34, 36, 37] reported the diagnostic tools utilised to determine the disease activity. Five studies [29, 31–34] used the Mayo score. Using that score, our analysis indicated significantly higher RDW levels in patients suffering from active UC (MD = 1.2; 0.63–1.78; *P* < 0.001). However, a significant heterogeneity was detected upon running the analysis (I^2 = 84%, *P* < 0.001). Two studies [13, 36] used Truelove and Witts’ criteria. Similarly, our calculations demonstrated significantly higher RDW levels in patients suffering from active UC (MD = 0.66; 0.33-1.0; *P* < 0.001 with insignificant heterogeneity (I^2 = 0%, *P* = 0.49). One study [37] utilised the Rachmilewitz Index (< 4 remission; ≥4 active) and demonstrated significantly higher RDW levels in patients suffering from active UC (MD = 1.3; 0.82–1.78; *P* < 0.001). The plot can be visualised in Fig. 15.

##### Patient stratification according to the UC disease activity Vs patients in remission

**Fig. 16:**
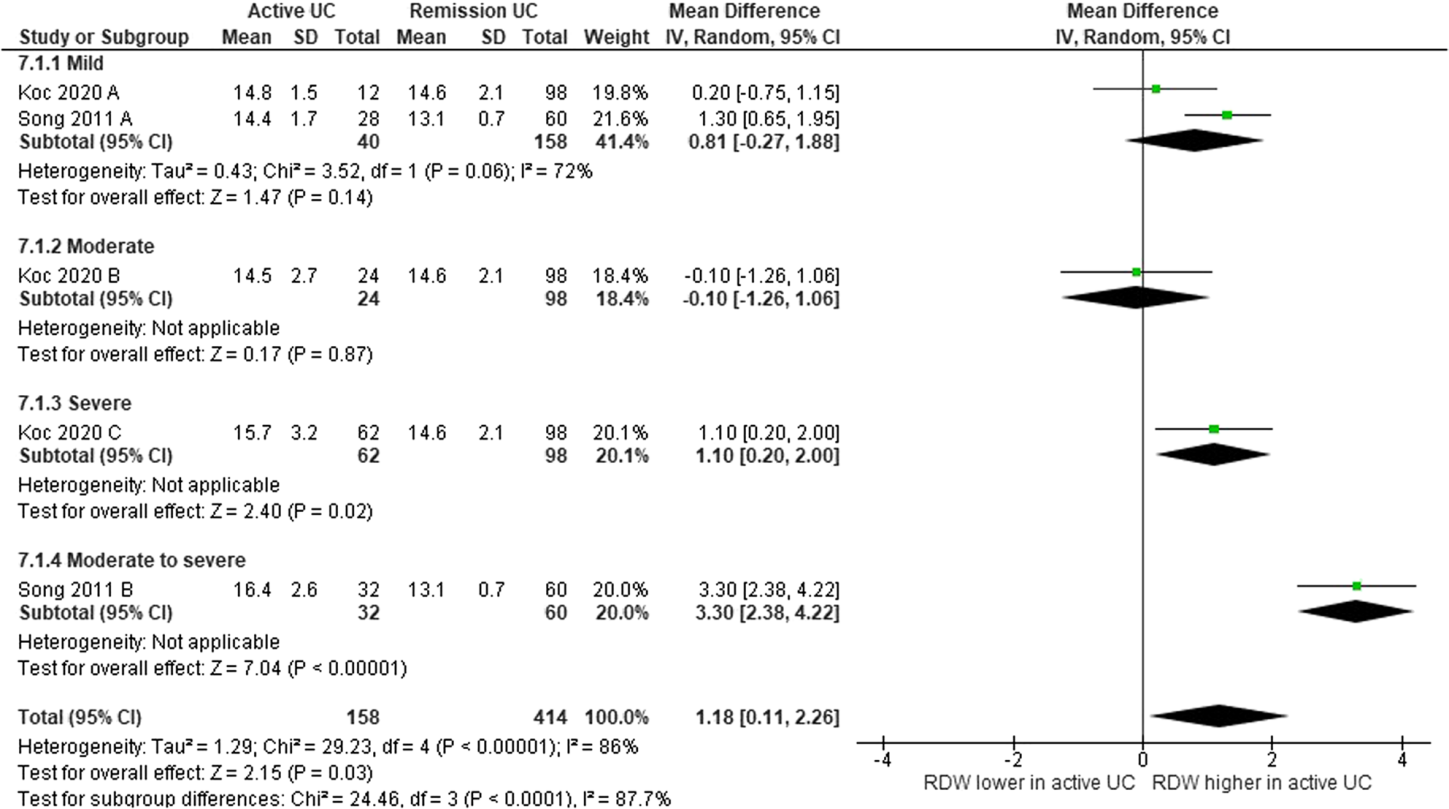
Patient stratification according to the UC disease activity Vs patients in remission

Two studies [32, 33] reported RDW values in patients with mild UC activity vs. levels in patients in remission, and the results were insignificant (*P* = 0.14). One study [32] reported RDW values in patients diagnosed with moderate and severe UC activity vs. levels in patients with UC in remission. In this study, the RDW levels in patients with moderate UC were not statistically significant (*P* = 0.87), However, significant RDW levels in patients with severe UC were demonstrated (MD = 1.1; 0.2- 2.0; *P* = 0.02). One study [33] indicated significant levels of RDW in patients with moderate to severe UC compared to patients in remission (MD = 3.3; 2.38–4.22; *P* < 0.001). The forest plot can be demonstrated in Fig. 16.

#### Diagnostic accuracy of RDW levels in ulcerative colitis

We pooled five studies [13, 33, 35–37] reporting on RDW diagnostic accuracy in differentiating between active and UC in remission. The disease activity was defined according to the Truelove–Witts criteria, Mayo score, or Rachmilewitz Activity Index. Our calculations demonstrated the sensitivity and the specificity of RDW in detecting UC severity as 0.53 (0.48–0.58) and 0.85 (0.8–0.89), respectively. The pooled DOR was 8.12 (3.77–17.51), while the PLR and NLR were 3.41 (2.28–5.09) and 0.46 (0.28–0.74), respectively; the AUC was calculated as 0.85 as demonstrated in Fig. 8.

Different studies used different RDW cut-off levels to identify active UC from UC in remission, with cut-off values ranging from 13.8% to 16.66%. Two studies [33, 35] utilised (≤ 14) as the RDW value cut-off to differentiate active UC from remission. As such, sensitivity and specificity were calculated as 0.82 (0.73–0.89) and 0.81 (0.72–0.89), respectively. The other studies [13, 36, 37] utilised (> 14) as the RDW cut-off, and that yielded sensitivity, specificity, and AUC of 0.44 (0.38–0.5), 0.87 (0.81–0.92), and 0.78, as demonstrated in Fig. 9.

### RDW levels in active CD patients versus RDW levels in active UC patients

**Fig. 17:**
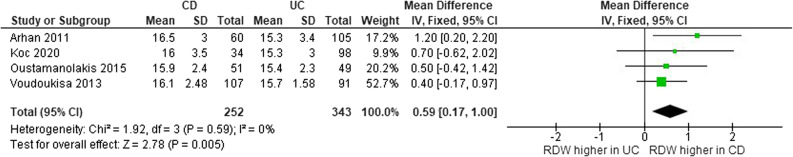
Comparison of RDW levels between patients with active Crohn’s disease and patients with active ulcerative colitis

Four studies [15, 24, 28, 32] compared the RDW levels in patients with active CD with the RDW levels in patients suffering from active UC. Interestingly, the RDW levels were significantly higher in patients with active CD (MD = 0.59; 0.17-1.0; *P* < 0.005) with insignificant heterogeneity (I^2 = 0%, *P* = 0.59), as shown in Fig. 17. One study [15] used an RDW cut-off of 14.45 to differentiate CD from UC. They demonstrated sensitivity, specificity, and AUC of 70%, 56%, and 0.67, respectively.

### Methodological quality assessment (AMSTAR 2)

Our evaluation demonstrated compliance with 15 out of the 16 domains, corresponding to an overall adherence rate of 94%, with compliance to all critical domains. According to commonly cited Interpretation thresholds, a score above 70% reflects high methodological rigor. According to AMSTAR 2 guidance, this corresponds to a rating of high confidence in the results of the present review. Accordingly, the synthesized evidence can be considered credible and reliable, as per Table 5.

**Table 5:** AMSTAR 2 – appraisal tool for systematic reviews including non-randomized studies

No.	AMSTAR 2 Item	Rating (Yes / Partial /No)
1	Did the research questions and inclusion criteria include the components of PICO?	Yes
2	Did the review have an explicit statement that the methods were established prior to the conduct of the review (e.g., a protocol)?	Yes
3	Did the review authors use a comprehensive literature search strategy?	Yes
4	Did the review authors perform study selection in duplicate?	Yes
5	Did the review authors perform data extraction in duplicate?	Yes
6	Did the review authors provide a list of excluded studies and justify the exclusions?	Yes
7	Did the review authors describe the included studies in adequate detail?	Yes
8	Did the review authors use a satisfactory technique for assessing the risk of bias (RoB) in individual studies?	Yes
9	Did the review authors report on the sources of funding for the studies included in the review?	No
10	If meta-analysis was performed, did the review authors use appropriate methods for statistical combination of results?	Yes
11	If meta-analysis was performed, did the review authors assess the potential impact of RoB in individual studies on the results?	Yes
12	Did the review authors account for RoB in individual studies when interpreting/discussing the results of the review?	Yes
13	Did the review authors provide a satisfactory explanation for, and discussion of, any heterogeneity observed in the results?	Yes
14	If they performed quantitative synthesis, did the review authors carry out an adequate investigation of publication bias?	Yes
15	Did the review authors report any potential sources of conflict of interest, including any funding they received for conducting the review?	Yes
16	Were the review authors’ conclusions supported by the results and did they consider the limitations of the included studies?	Yes

### GRADE assessment of evidence quality

We assessed our main outcomes using the GRADE assessment; overall, the certainty ranged from High to low. RDW values to distinguish active CD from active UC were rated as high. The comparison between CD activity versus healthy control, active CD versus remission, CD activity vs. remission, UC activity vs. healthy control, and UC activity versus remission was downgraded by one point due to inconsistency (high heterogeneity). On the other hand, the comparison between CD versus healthy control, UC versus healthy control, and active UC versus remission was downgraded by two points due to inconsistency and probable publication bias demonstrated in supplementary Figs. 3–6. These results indicate that the real effect of RDW values in comparison to IBD is likely similar to what we found, but it could still turn out to be quite different, as shown in Table 6.

**Table 6:** GRADE assessment

Outcome	No. of studies	Study design	Risk of Bias	Inconsistency	Indirectness	Imprecision	Publication bias	95% CI	grade score	Certainty
RDW values in CD versus healthy	9	observational	Low	Serious	Not serious	Not serious	Serious	1.6–2.79	-2	Low
RDW values in patients with active CD compared with healthy controls	5	Observational	Low	Serious	Not serious	Not serious	Not serious	1.46–2.85	-1	Moderate
RDW values in overall active CD versus remission	9	observational	Low	Serious	Not serious	Not serious	Not serious	0.94–1.65	-1	Moderate
RDW values in severity-stratified CD activity versus remission	3	Observational	Low	Serious	Not serious	Not serious	Not serious	0.81–2.57	-1	Moderate
RDW values in UC versus healthy subject	5	observational	Low	Serious	Not serious	Not serious	Serious	0.72-2	-2	Low
RDW values in patients with active UC compared with healthy controls	2	observational	Low	Serious	Not serious	Not serious	Not serious	0.26–1.27	-1	Moderate
RDW values in overall active UC versus remission	8	observational	Low	Serious	Not serious	Not serious	Serious	0.71–1.5	-2	Low
RDW values in severity-stratified UC activity versus remission	2	observational	Low	Serious	Not serious	Not serious	Not serious	0.11–2.26	-1	Moderate
Comparison of RDW values in patients with active CD and patients with active UC	4	observational	Low	Not Serious	Not serious	Not serious	Not serious	0.17-1.0	0	High

## Discussion

In this systematic review and meta-analysis, we synthesized evidence to evaluate the practical utility of red cell distribution width (RDW) in inflammatory bowel disease (IBD), including both Crohn’s disease (CD) and ulcerative colitis (UC). Our analysis, which included nineteen studies with a pooled number of 3,780 participants, highlights that RDW rises in patients suffering from IBD compared to healthy individuals. We observed significantly higher levels of RDW during active disease states compared to remission. As a result, this review indicates that we may use RDW as a simple, available biomarker for the detection and evaluation of disease activity in IBD.

Throughout the included studies, RDW levels were found to be significantly higher in patients with CD when compared to healthy controls, with a mean difference of 2.2. Additionally, higher values were detected in those suffering from active disease compared to remission. In relation to UC, similar results were observed with significantly higher levels in patients suffering from UC compared to healthy individuals. Similarly, higher levels were detected in active UC disease compared with remission. These results, consistently observed in both disease phenotypes, highlight that RDW can potentially be utilised as a strong indicator of systemic inflammation in IBD. Among the included studies, an RDW threshold of > 14% was the most frequently utilized cut-off for differentiating active Crohn’s disease from remission. Pooled analysis of these studies demonstrated a sensitivity of 0.86 and a specificity of 0.78 with an AUC of 0.83, suggesting that this threshold may serve as a practical reference value [14, 22, 30, 33, 38]. 

The observed increase in RDW levels among the patients suffering from IBD can be attributed to the combined effect of chronic inflammation, in addition to anaemia of chronic disease. Inflammatory cytokines can lead to shorter erythrocyte life spans by interfering with erythropoiesis. That in turn results in anisocytosis and elevated RDW [30, 33]. Furthermore, the malabsorption of certain nutrients, such as iron, vitamin B12, and folate, plays some role in iron-restricted erythropoiesis [30]. As a result of such multifactorial pathophysiology, RDW’s sensitivity to both inflammatory and hematinic disturbances in IBD can be explained.

Our results also indicated that RDW levels were significantly higher in patients with active CD compared with active UC. One study reported that a cut-off of 14.45% can distinguish between the two diseases with a 70% sensitivity and 56% specificity [15, 32, 33]. Although RDW levels were significantly higher in patients with active CD compared with active UC, the observed effect size was modest. This suggests that, despite statistical significance, the between-group difference may have limited clinical relevance when used in isolation. However, it may assist in solving the diagnostic challenges that usually present when the differentiation is solely based on clinical and endoscopic features.

The potential clinical use of RDW can be further supported by its diagnostic accuracy. The pooled area under the ROC curve (AUC) for RDW to detect patients with active CD was 0.8, and 0.85 for UC. That indicates a reliable diagnostic performance [14, 26, 30, 33]. While RDW demonstrated higher sensitivity in CD patients, it showed greater specificity in UC patients. These results can be attributed to the variation in inflammatory patterns and iron metabolism between the disease phenotypes. Putting into consideration that RDW testing is a cheap and widely available test that can be obtained through routine blood count, it can stand as a practical, cost-effective adjunct in IBD evaluation and follow-up.

Practically speaking, RDW cannot replace the already established gold standard diagnostic modalities, such as colonoscopy or imaging. However, it may be utilised as a complementary laboratory marker next to C-reactive protein (CRP) and erythrocyte sedimentation rate (ESR) [30]. Our analysis highlights that RDW correlated significantly with the disease activity. That supports the potential use for identifying subclinical inflammation in remission phases, early disease detection, and monitoring therapeutic response. Nevertheless, whether RDW offers additional clinical value beyond established biomarkers such as CRP, fecal calprotectin, albumin, ferritin, and hemoglobin remains unclear and requires further investigation in prospective studies.

Despite all the significant results, our study is not without limitations. Most of the included studies were observational in design, predominantly retrospective or cross-sectional, which are weaker than other study designs. Throughout our meta-analysis, significant heterogeneity was observed across the included studies. I² values reached up to 70% in some comparisons. That may be explained by differences in study design, disease duration, population characteristics, and the heavy reliance on clinical activity scoring systems (e.g., CDAI, Mayo score, Truelove–Witts), While these are common in clinical research, endoscopic evaluation is currently recognized as the gold standard for determining IBD activity and treatment response, as endorsed by ECCO guidelines [5, 7]. Clinical scores may misestimate true mucosal inflammatory activity due to symptom subjectivity. This difference between clinical and endoscopic activity likely contributed to heterogeneity. In addition, variability in laboratory methods, differences in RDW reference ranges, disparities in treatment status and medication use, and varying definitions of disease activity across studies may have further contributed to the observed heterogeneity. These methodological and clinical differences should be taken into consideration when interpreting the pooled estimates.

Furthermore, additional research is needed to better characterize the relationship between RDW and iron deficiency in IBD, as well as the potential influence of other factors known to affect RDW values, including nutritional deficiencies, anemia, and blood transfusions. This heterogeneity, in addition to publication bias, led to moderate grading of most comparisons using the GRADE assessment tool. Overall, the significant association across all our calculations supports the usage of RDW as an inflammatory biomarker in IBD, but the lack of consistent endoscopic correlation across all pooled studies may limit the precision of RDW in reflecting true biological remission versus clinical symptoms.

In our opinion, future studies should focus on establishing standardized RDW cut-off values, particularly for UC. Research should also explore the possibility of combining RDW with other biomarkers to formulate new diagnostic indices for both UC and CD. In addition, prospective studies should be conducted to assess RDW changes over time to further investigate its prognostic value.

## Conclusion

In conclusion, this meta-analysis demonstrates that RDW levels are significantly elevated in inflammatory bowel diseases and are consistently associated with disease activity. Given its wide availability and low cost, RDW may have value as an adjunctive laboratory parameter when interpreted alongside established inflammatory biomarkers and gold-standard endoscopic evaluation. Further prospective studies are needed to clarify its incremental diagnostic and prognostic role in clinical practice.

## Supplementary Information

Supplementary Material 1.

## Data Availability

All data analysed during this study are included in this published article and its supplementary information files.
