# Supplementary material for: The role of red cell distribution width in inflammatory bowel disease evaluation: a comprehensive systematic review and meta-analysis
Source: BMC Gastroenterol. 2026 Jul 27;26:475. doi: 10.1186/s12876-026-05156-y (PMC13412027; doi:10.1186/s12876-026-05156-y)
Supplement: Supplementary file 1 — Supplementary Material 1. [file 12876_2026_5156_MOESM1_ESM.docx]

| Database | Search strategy | Results Retrieved |
| --- | --- | --- |
| PubMed | (Anisocytosis OR red cell distribution width OR RDW OR red blood cell distribution width OR erythrocyte volume distribution width OR red cell volume distribution width) AND (inflammatory bowel disease OR ulcerative colitis OR Crohn disease OR Crohn’s disease OR Crohn’s enteritis OR regional enteritis OR ileocolitis OR terminal ileitis OR IBD OR CD OR UC) | 182 |
| Scopus |  | 410 |
| Web Of Science |  | 286 |
| Total | 878 | |

Supplementary table1: for search strategy and results


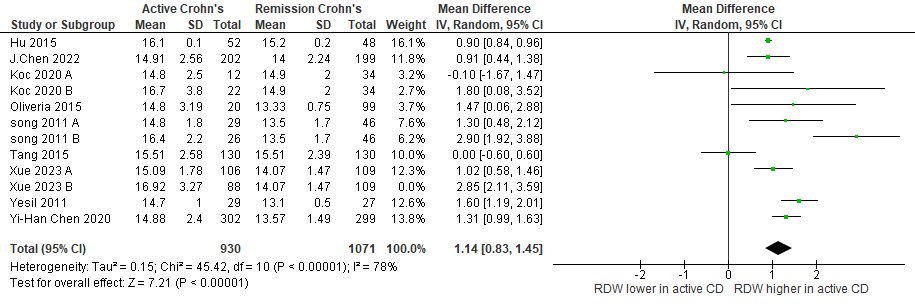


Supplementary figure 1: Leave one out: CD active vs remission (Xue B excluded)


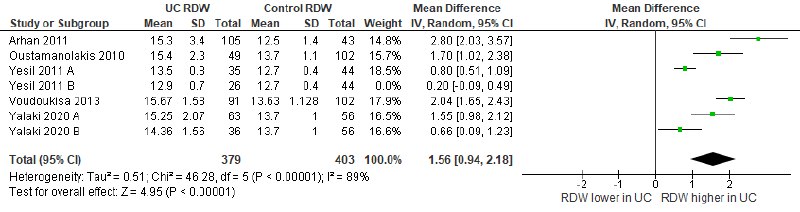


Supplementary figure 2: Leave one out: UC vs healthy (Yesil excluded)


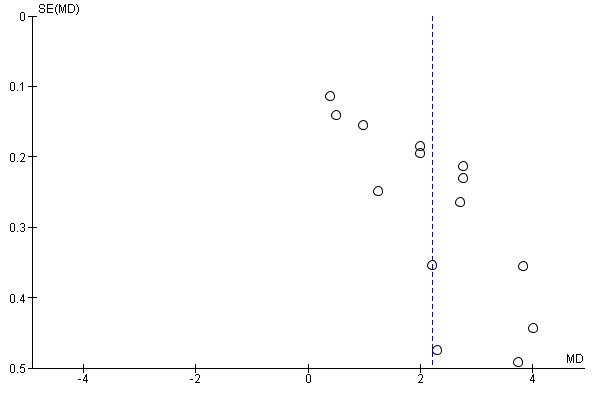


Supplementary figure 3: Funnel plot: CD vs Healthy


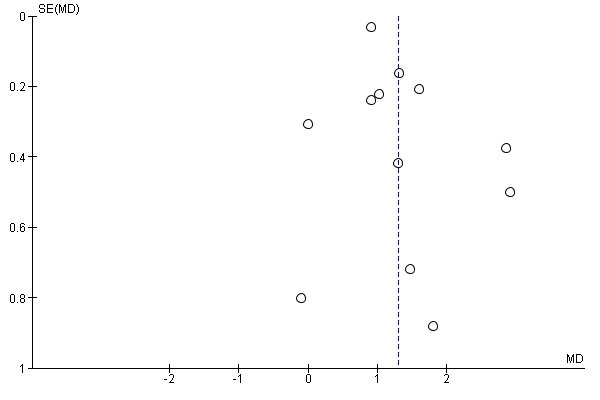
 Supplementary figure 4: Funnel plot: CD active vs remission


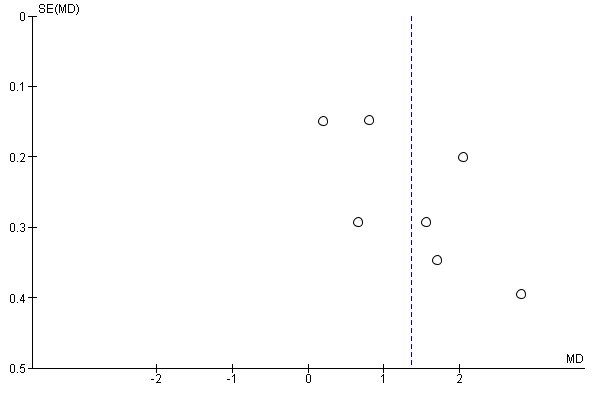


Supplementary figure 5: Funnel plot: UC vs healthy


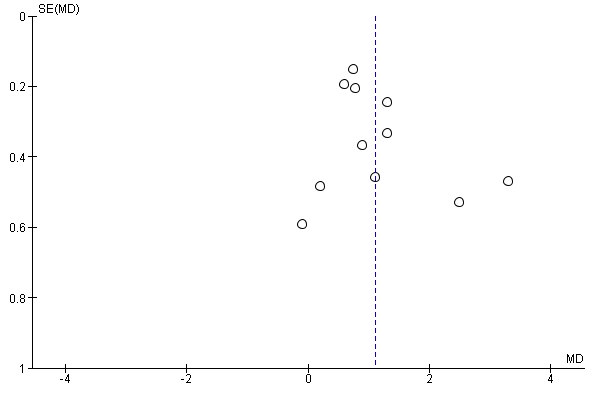


Supplementary figure 6: Funnel plot: UC active vs remission


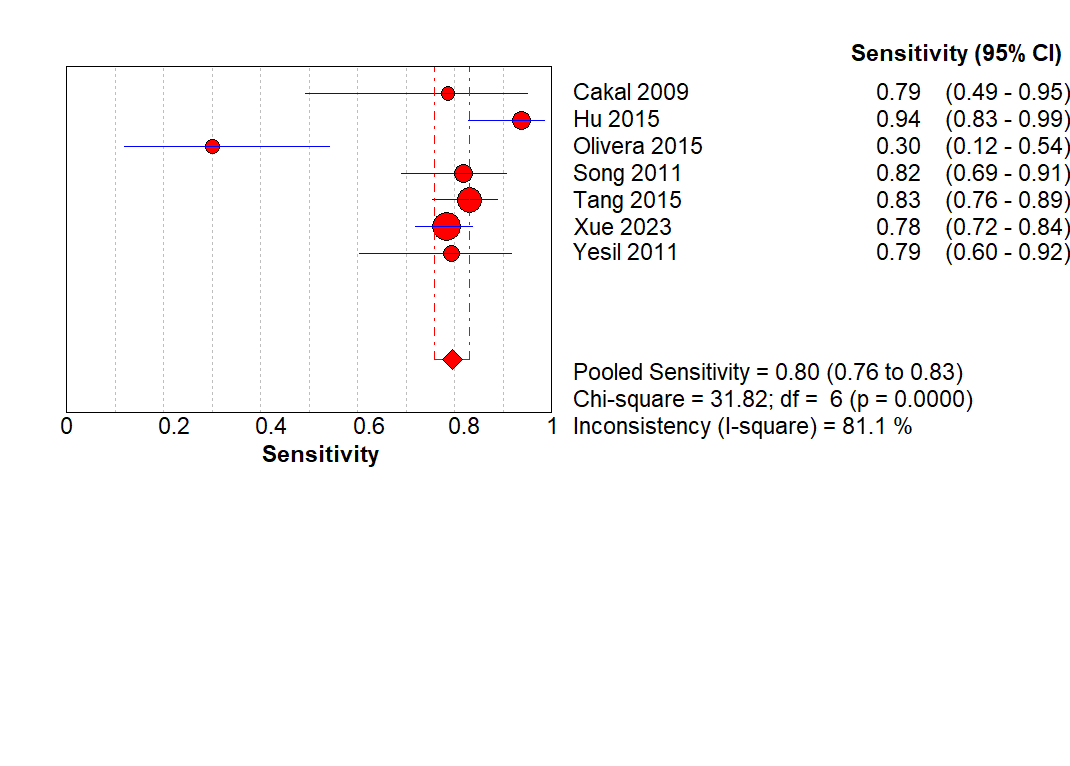


Supplementary figure 7: RDW sensitivity to detect CD activity


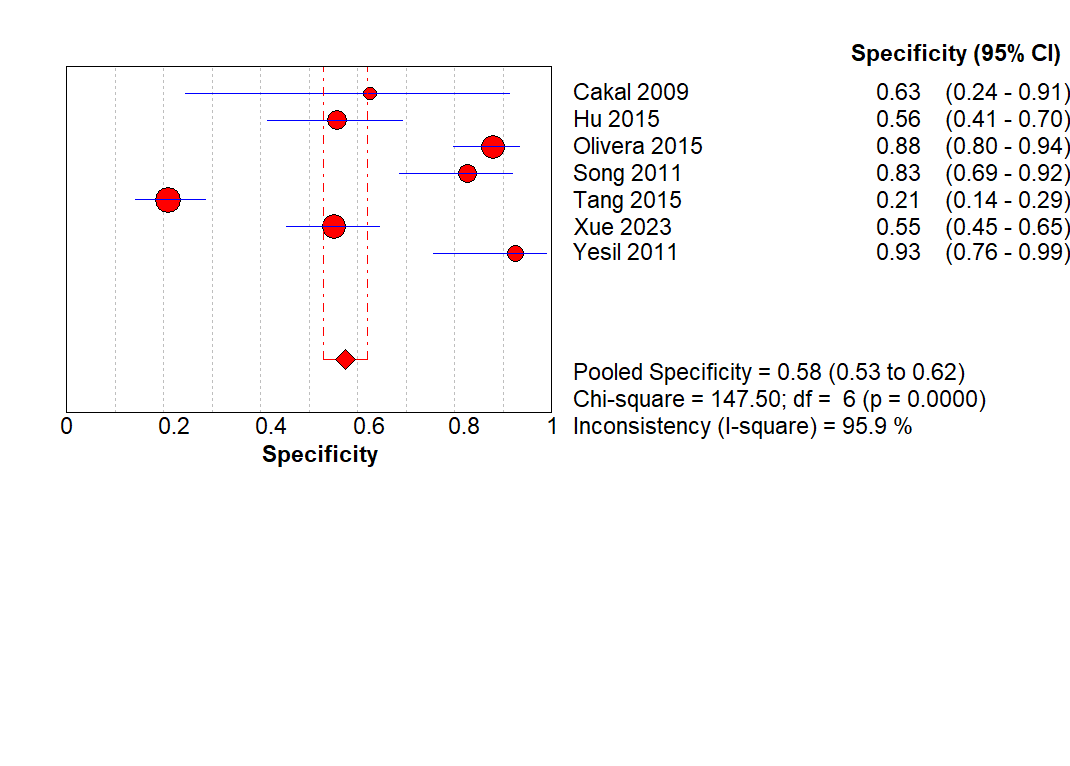


Supplementary figure 8: RDW specificity to detect CD activity


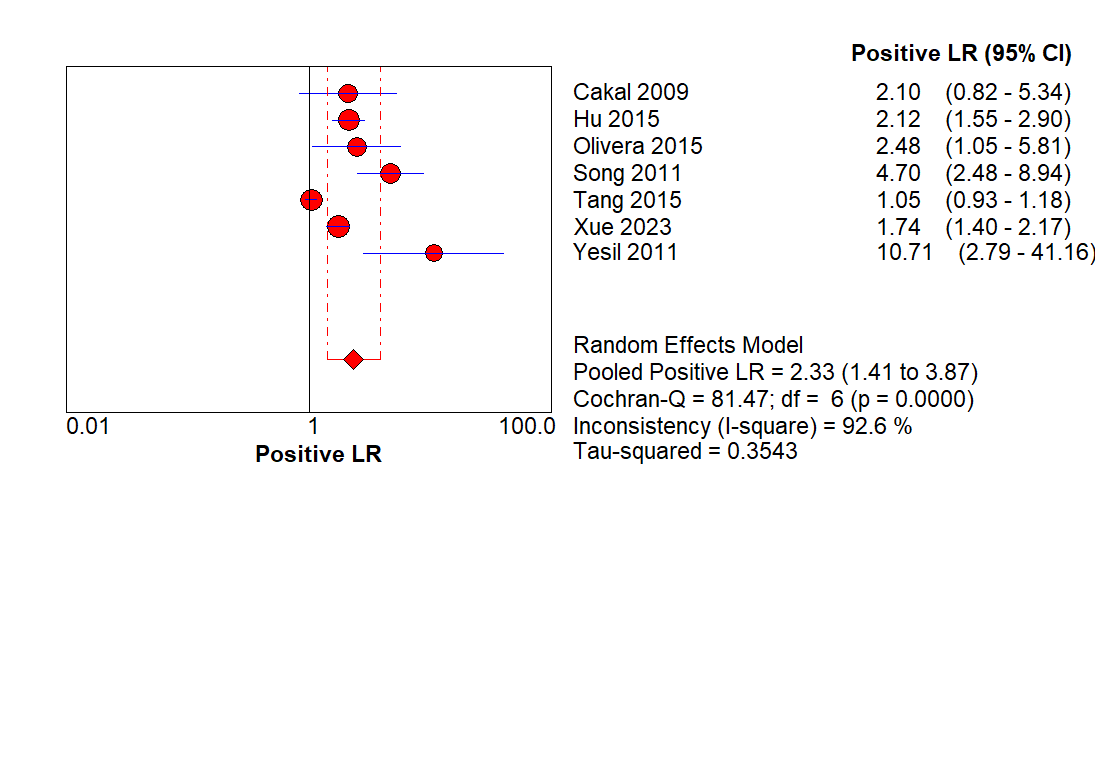


Supplementary figure 9: RDW positive likelihood ratio to detect CD activity


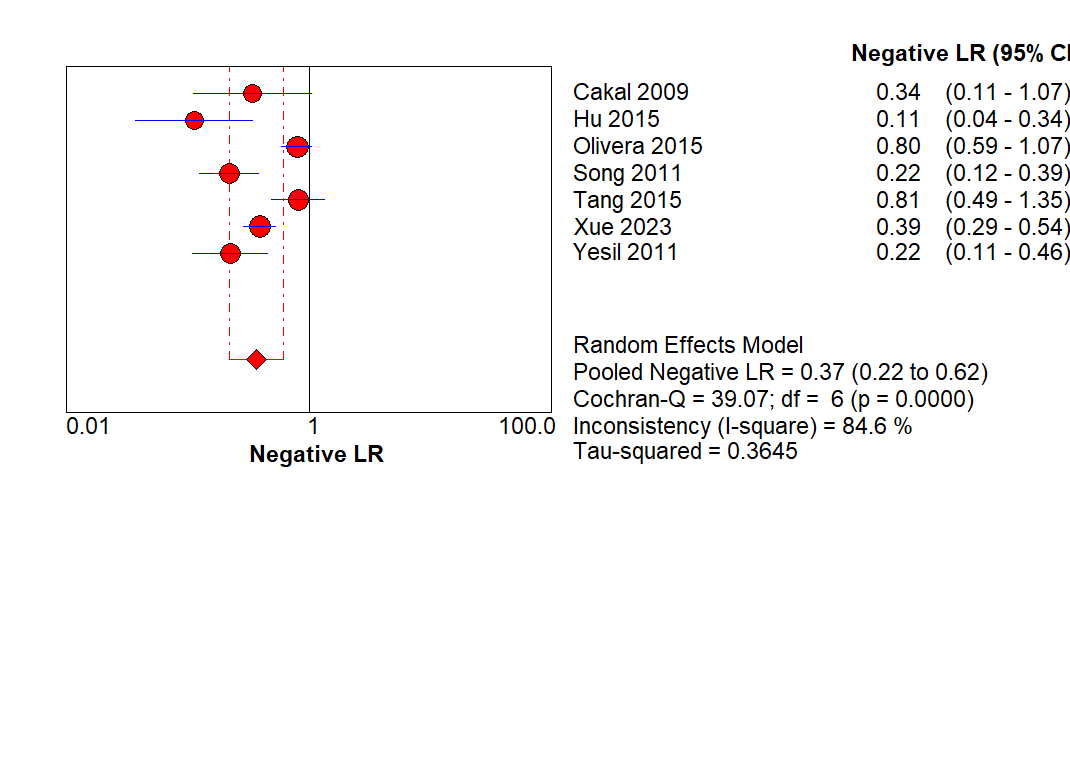


Supplementary figure 10: RDW negative likelihood ratio to detect CD activity


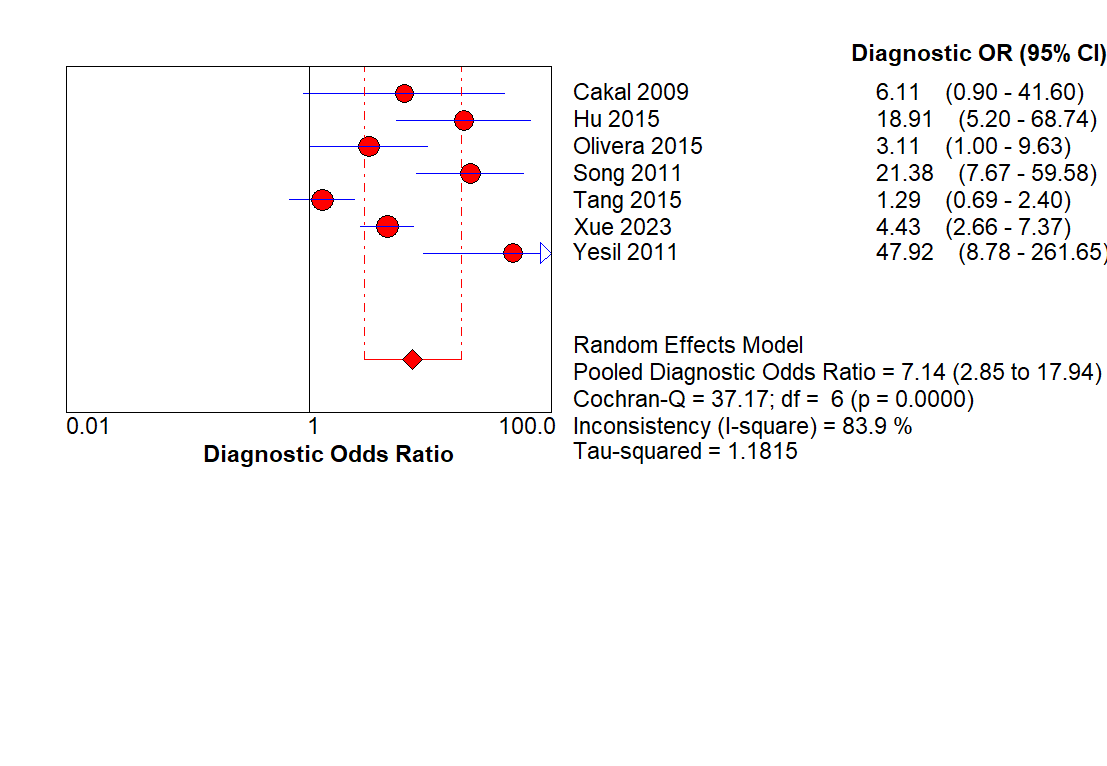


Supplementary figure 11: RDW diagnostic odds ratio to detect CD activity


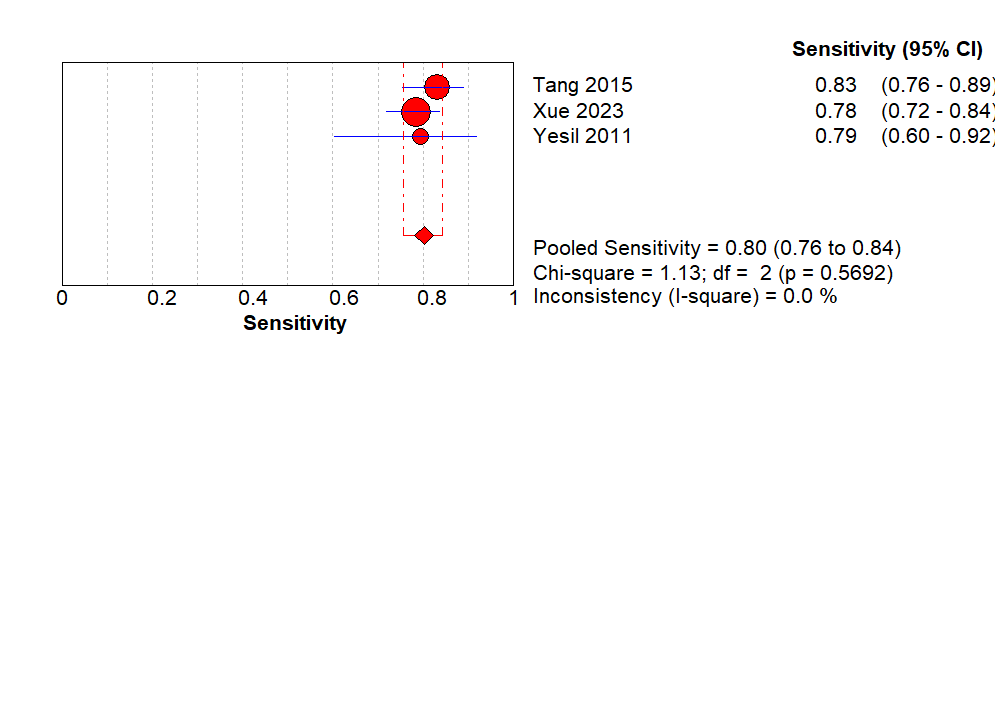


Supplementary figure 12: RDW 14 or less sensitivity to detect CD activity


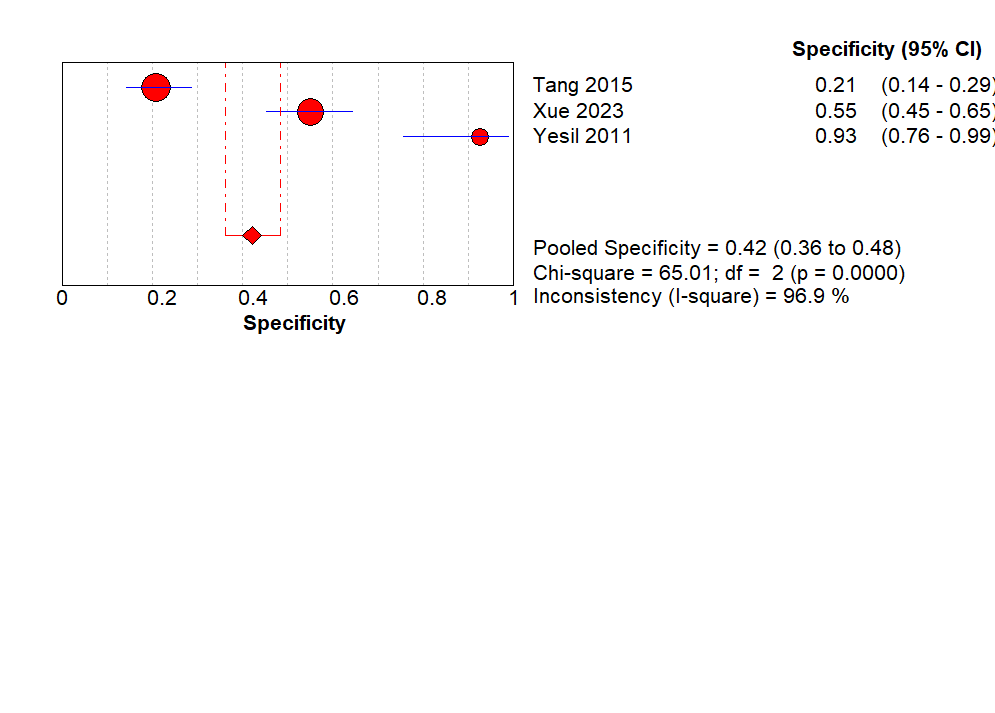


Supplementary figure 13: RDW 14 or less specificity to detect CD activity


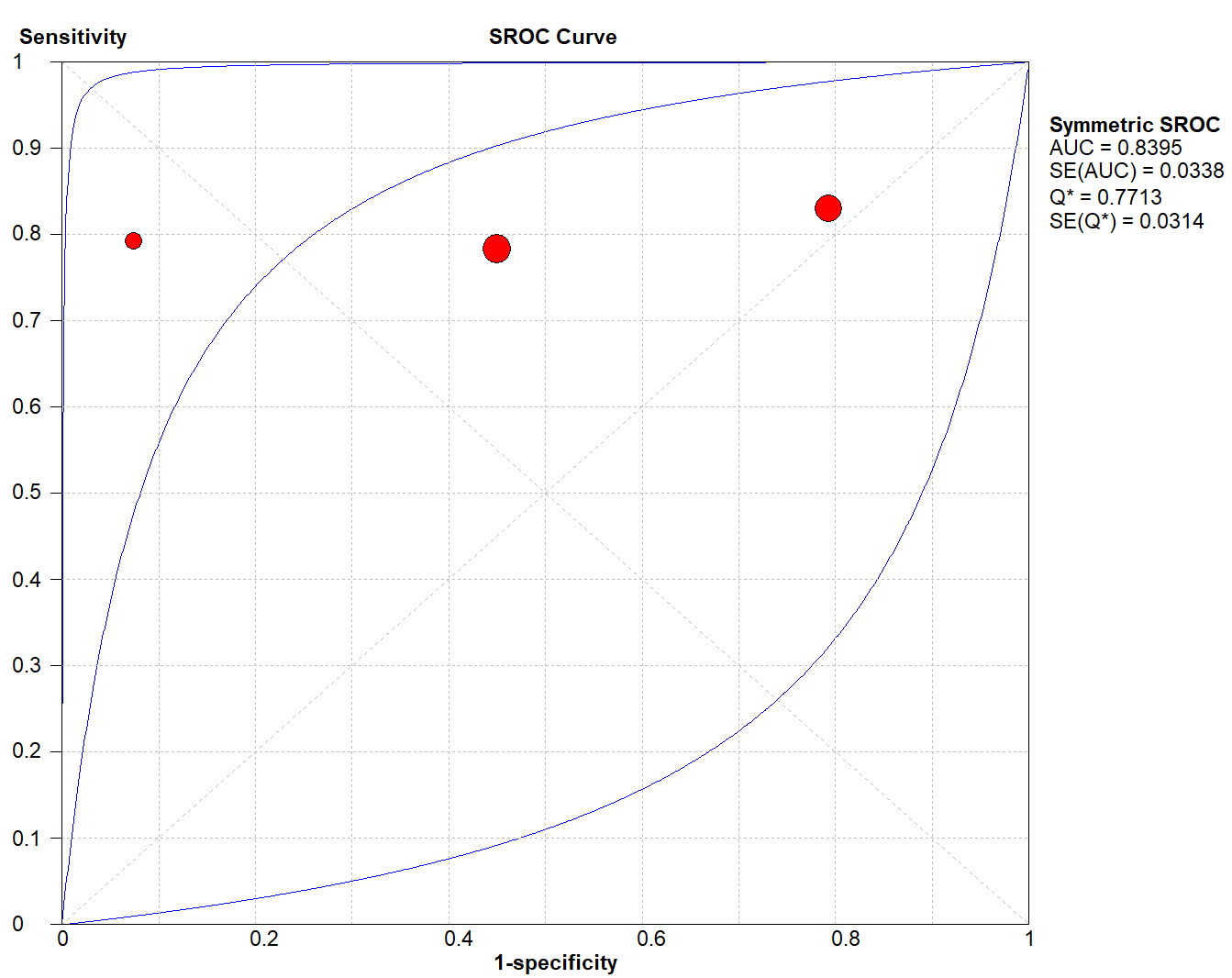


Supplementary figure 14: RDW 14 or less SROC to detect CD activity


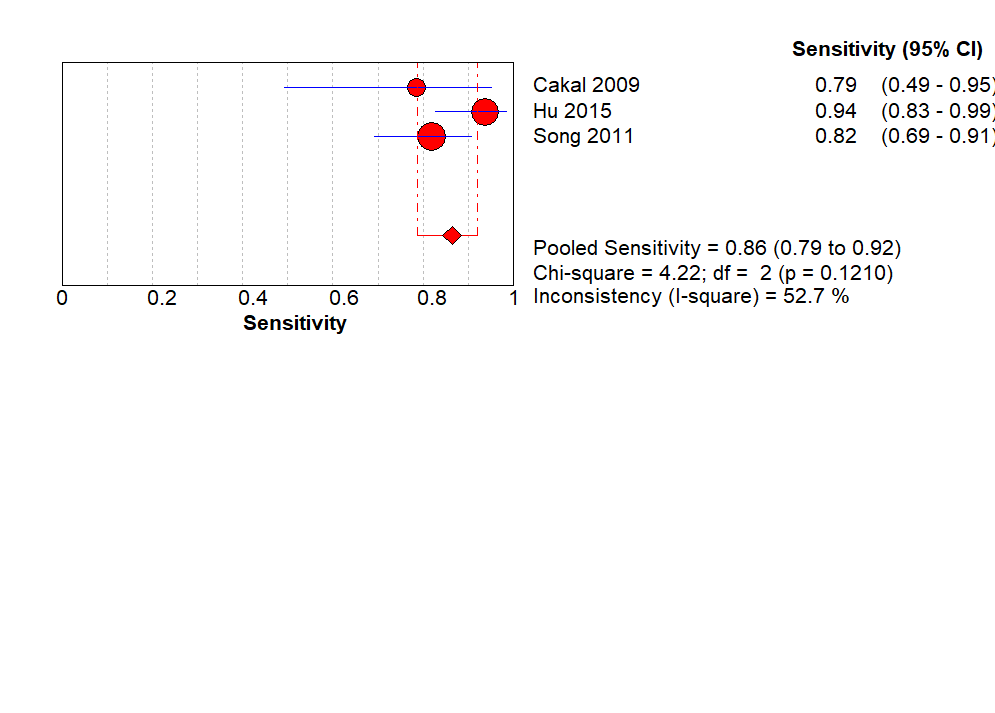


Supplementary figure 15: RDW more than 14 sensitivity (Olivera Excluded) to detect CD activity


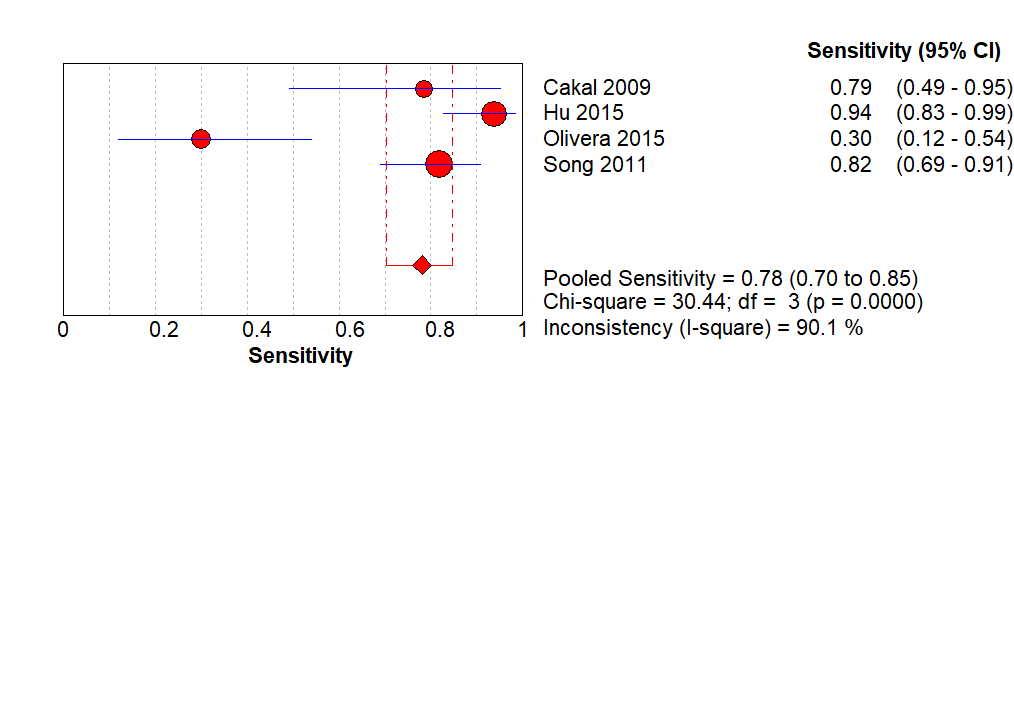


Supplementary figure 16: RDW more than 14 sensitivity to detect CD activity


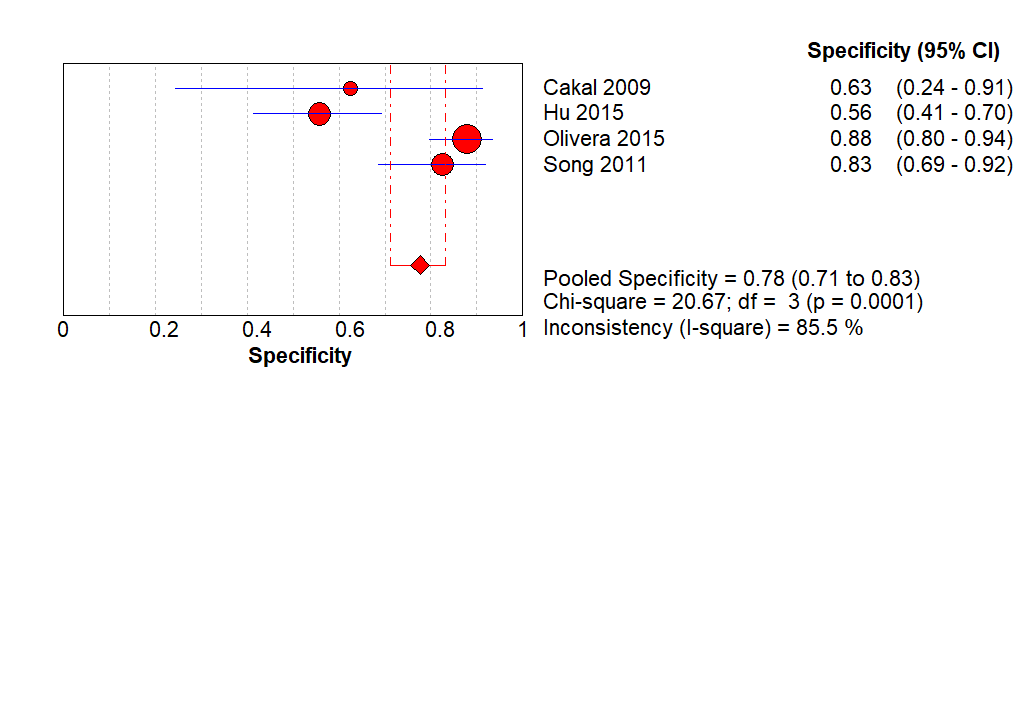


Supplementary figure 17: RDW more than 14 specificity to detect CD activity


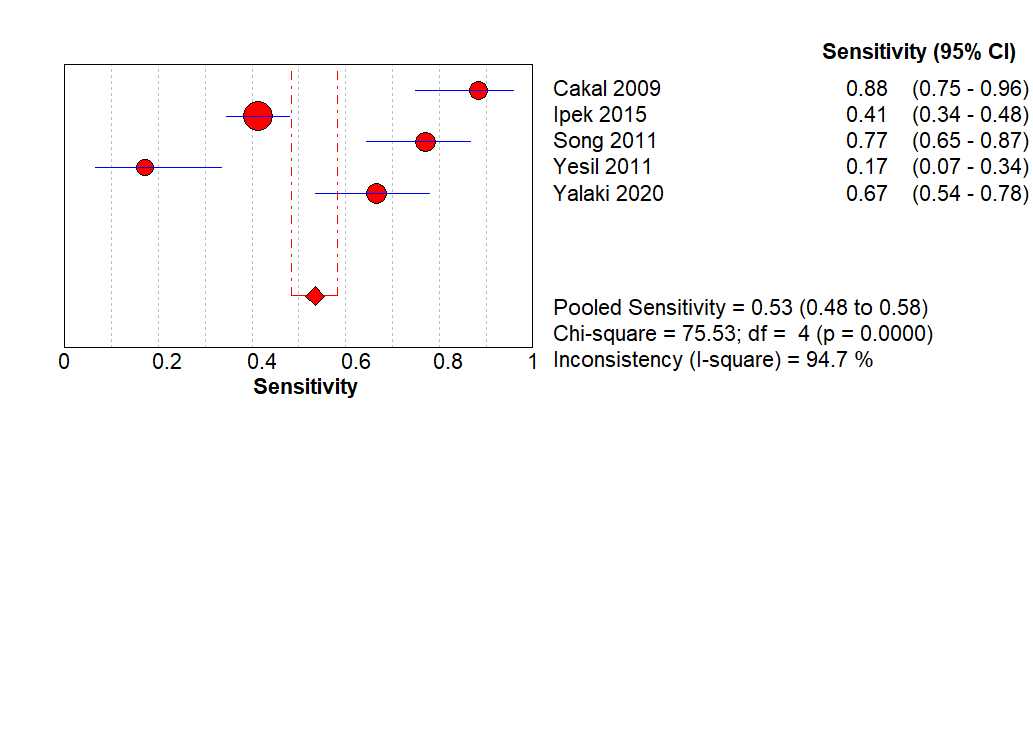


Supplementary figure 18: RDW sensitivity to detect UC activity


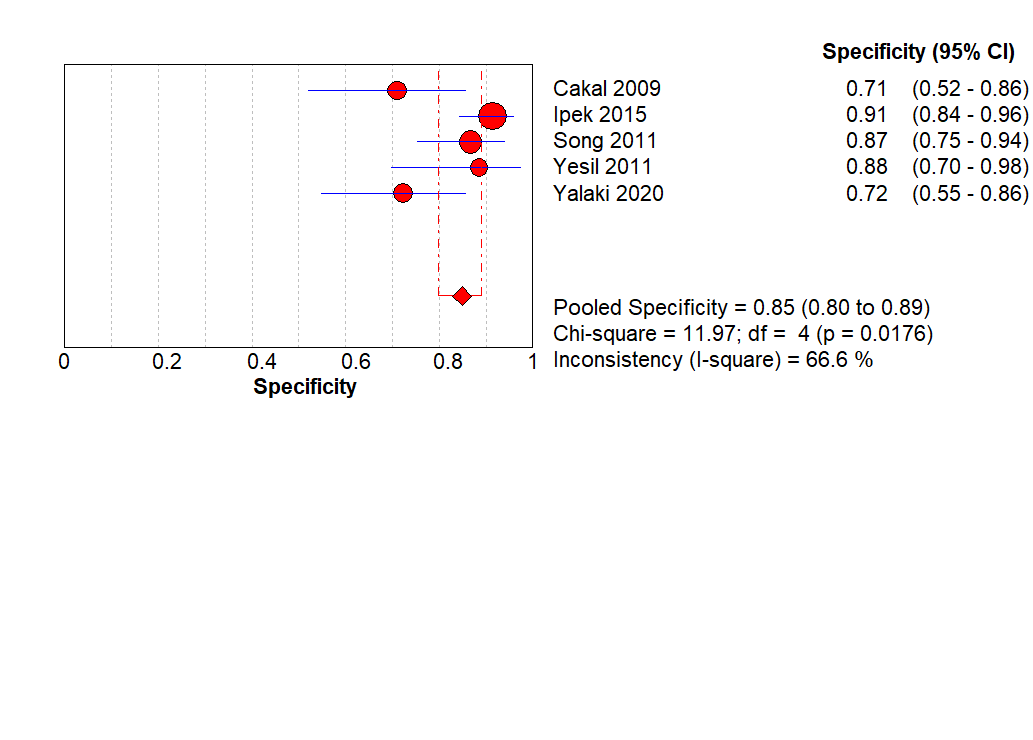


Supplementary figure 19: RDW specificity to detect UC activity


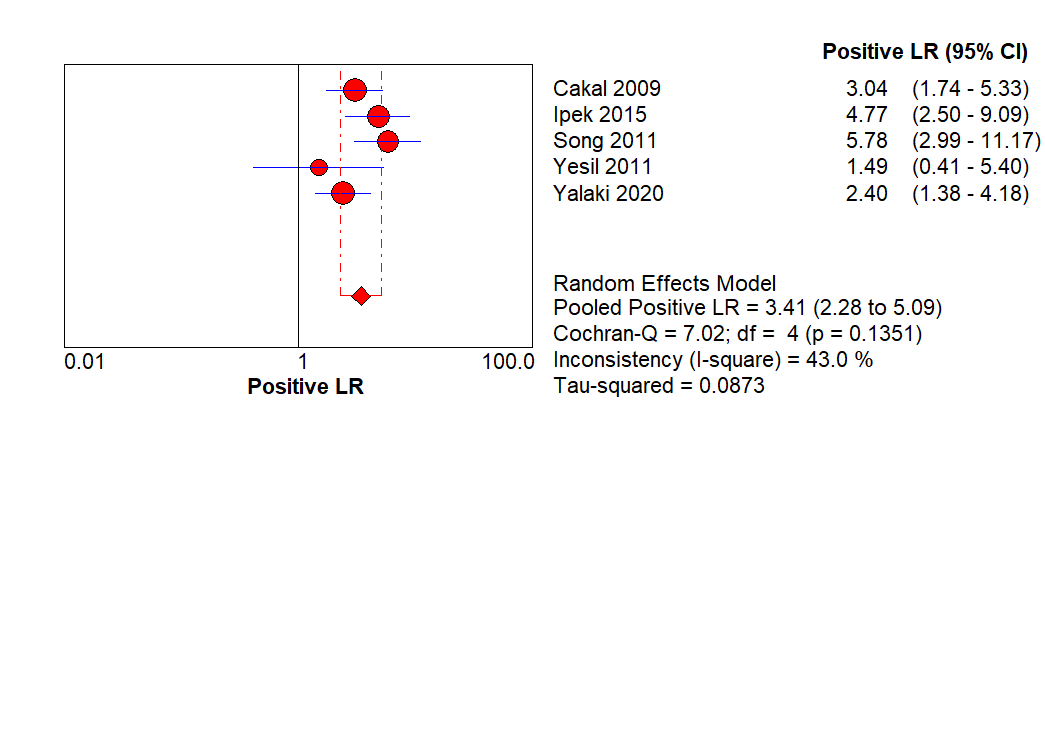


Supplementary figure 20: RDW positive likelihood ratio to detect UC activity


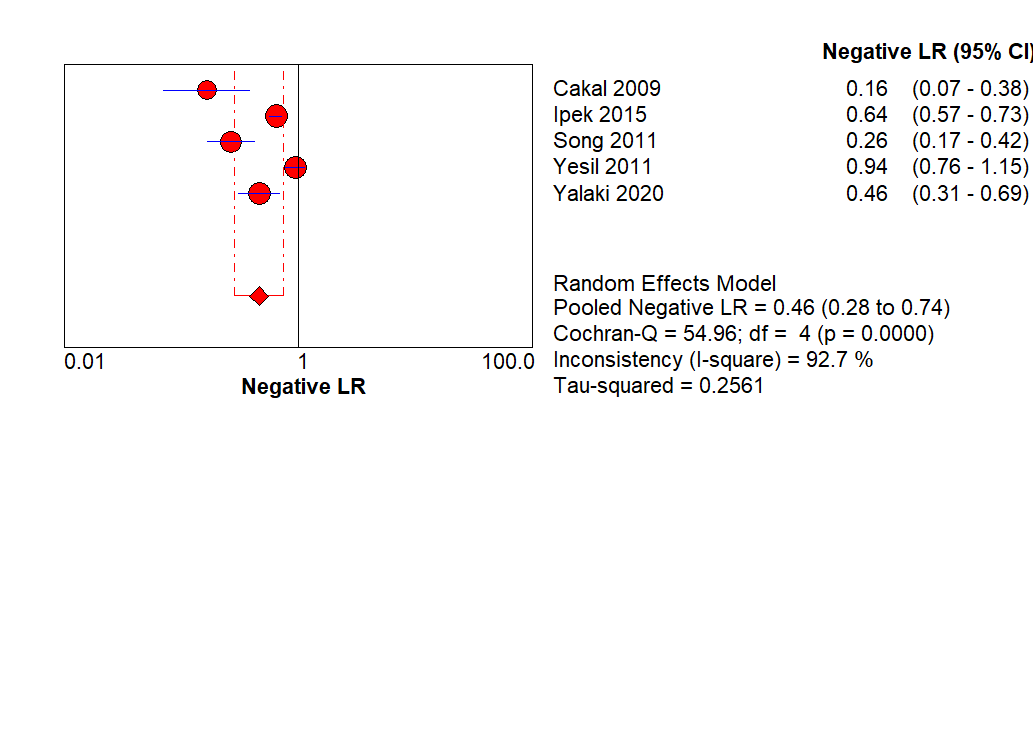


Supplementary figure 21: RDW negative likelihood ratio to detect UC activity


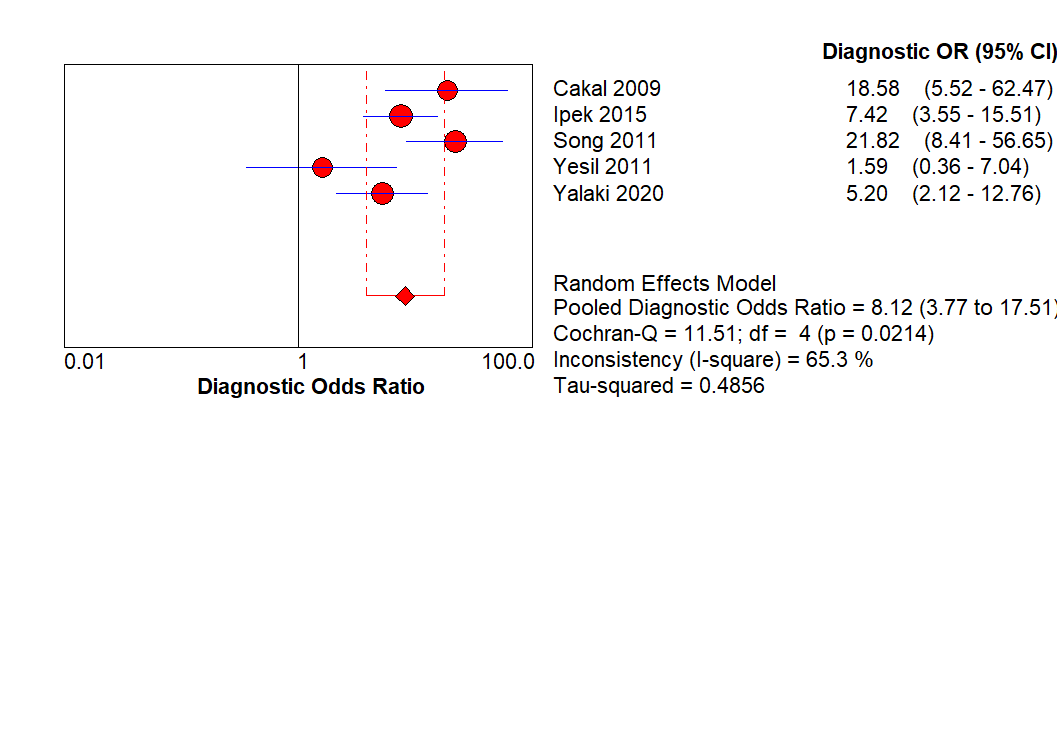


Supplementary figure 22: RDW diagnostic odds ratio to detect UC activity
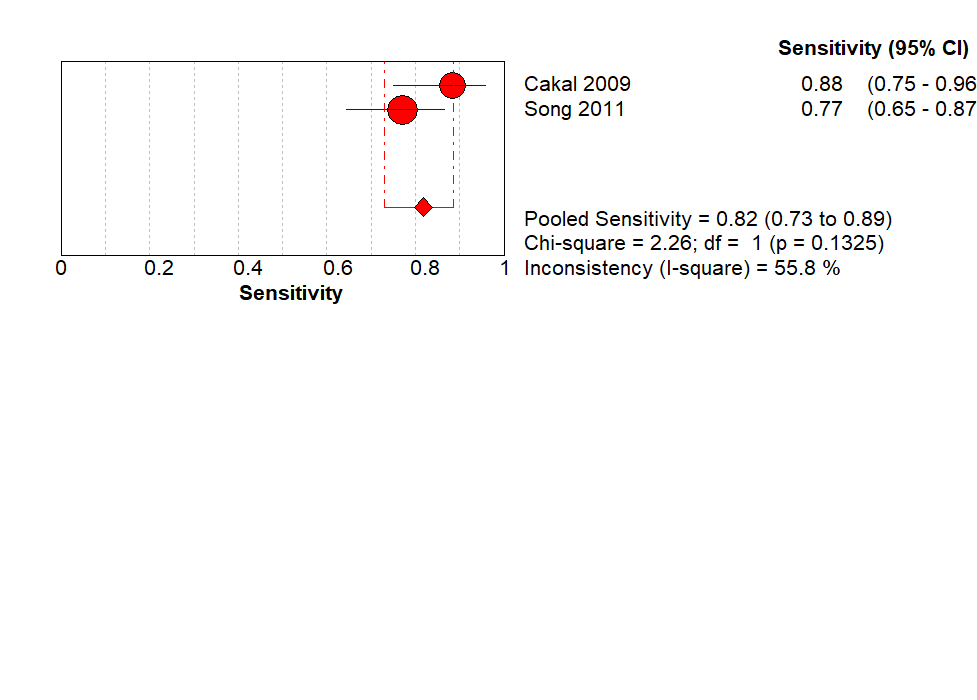


Supplementary figure 23: RDW 14 or less sensitivity to detect UC activity


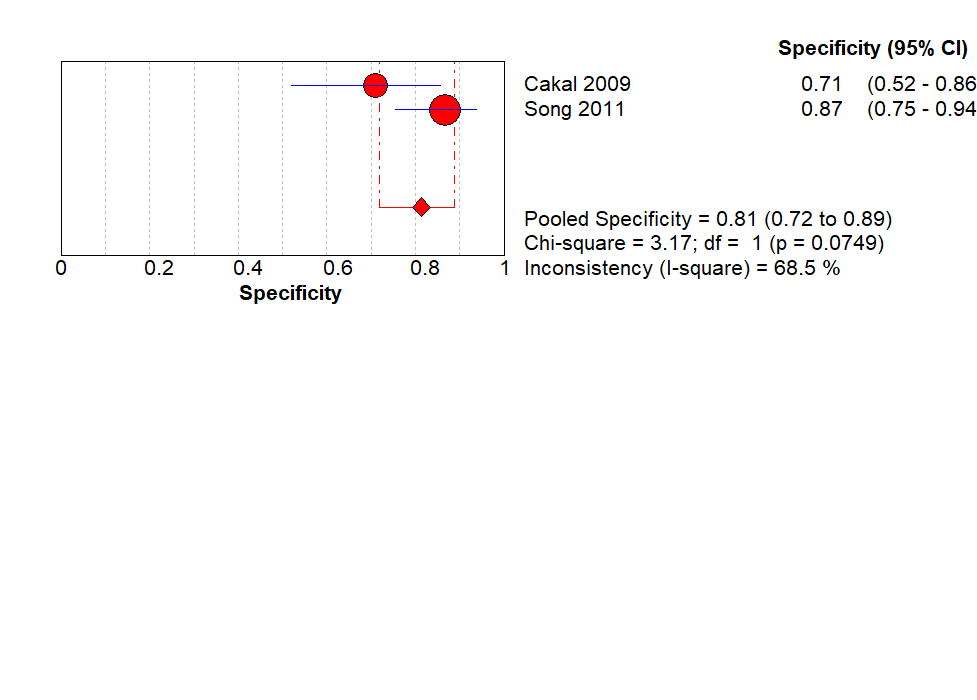


Supplementary figure 24: RDW 14 or less specificity to detect UC activity


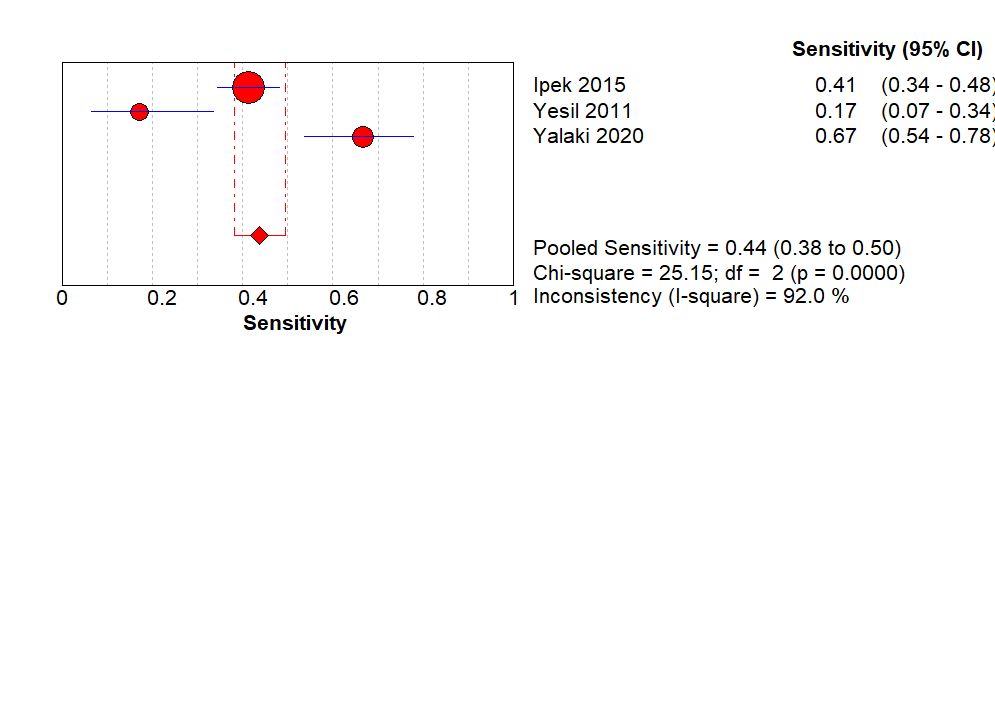


Supplementary figure 25: RDW more than 14 sensitivity to detect UC activity


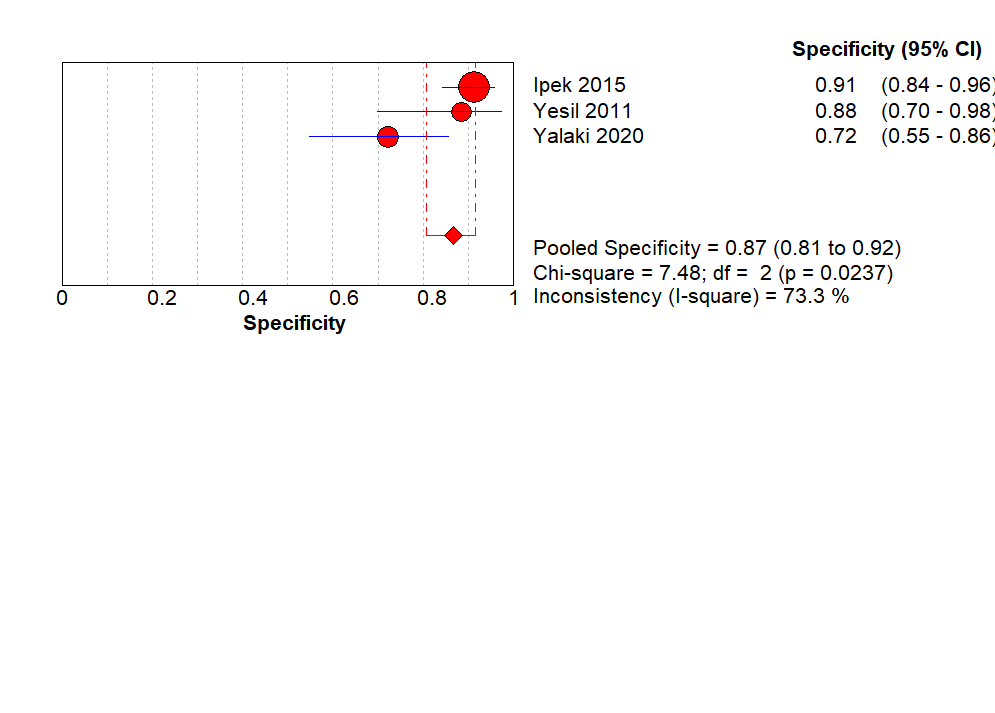


Supplementary figure 26: RDW more than 14 specificity to detect UC activity
